# Supplementary material for: 4D Single-particle tracking with asynchronous read-out single-photon avalanche diode array detector
Source: Nat Commun. 2024 Jul 23;15:6188. doi: 10.1038/s41467-024-50512-9 (PMC11266502; doi:10.1038/s41467-024-50512-9)
Supplement: Supplementary file 1 — Supplementary Information [file 41467_2024_50512_MOESM1_ESM.pdf]

SUPPLEMENTARY INFORMATION NOTES

SI Note 1 Cramér-Rao bound derivation

In order to calculate the Cramér-Rao bound (CRB), we adapt the mathematical framework described in [1, 2] by introducing a structured detection.

We begin by considering a fluorophore in position $\mathbf{r}_\mathrm{e}$ providing $\mathbf{n} = (n_1, n_2, n_3, ..., n_K)$ photon countings for the $K = 25$ different single-photon avalanche diode (SPAD) array detector elements. The total number of photons detected, denoted as N, is given by the sum:

$$N = \sum_{i=1}^{K} n_i$$

We assume each counting n_i to follow an independent Poisson distribution $n_i \sim \mathrm{Poisson}(\lambda_i)$ with expected value λ_i. The distribution of all the countings $\mathbf{n}$ conditioned to N is described by a Multinomial distribution. Consequently, we can write the likelihood function of the emitter position $\mathbf{r}_\mathrm{e}$ as:

$$\mathcal{L}(\mathbf{r}_\mathrm{e}|\mathbf{n}) = \frac{N!}{\prod_{k=1}^{K} n_k!} \cdot \prod_{k=1}^{K} p_k(\mathbf{r}_\mathrm{e})^{n_k} \tag{S.1}$$

where $p_k(\mathbf{r}_\mathrm{e})$ is the probability that a detected photon belongs to the k-th element. Therefore, each multinomial parameter is calculated as:

$$p_k(\mathbf{r}_\mathrm{e}) = \frac{\lambda_k(\mathbf{r}_\mathrm{e})}{\sum_{i=1}^{K} \lambda_i(\mathbf{r}_\mathrm{e})} \tag{S.2}$$

We start by examining a scenario where there's no background, which we denote with the superscript (0). All detected photons originate from the single particle, therefore by defining $s_i(\mathbf{r}_\mathrm{e})$ the expected value of the signal, we write:

$$\lambda_i^{(0)}(\mathbf{r}_\mathrm{e}) = s_i(\mathbf{r}_\mathrm{e}) \tag{S.3}$$

Given a certain number N_exc of excitation photons that reaches the sample, the detected signal then depends on the probability of effectively exciting the particle $P(\mathrm{excitation})$ and subsequently detecting an emitted fluorescent photon on the i-th element $P_i(\mathrm{detection}|\mathrm{excitation})$:

$$s_i = N_\mathrm{exc} \cdot P(\mathrm{excitation}) \cdot P_i(\mathrm{detection}\,|\,\mathrm{excitation}) \tag{S.4}$$

If we assume both the excitation intensity and the detection rate of the SPAD array are far from saturation, then we can model each parameter of Eq. S.4 with a linear relation

$$
\begin{cases}
N_{\text{exc}} & = \ \phi_{\text{exc}} \cdot \Delta t \\
P(\text{excitation}) & = \ h_{\text{exc}}(\mathbf{r}_{\text{e}}) \cdot \sigma_{\text{abs}} \\
P_i(\text{detection} \,|\, \text{excitation}) & = \ \Phi_{\text{fl}} \cdot \eta_{\text{det}} \cdot \displaystyle\int_{\mathbb{R}^3} h_{\text{em}}(\mathbf{r} - \mathbf{r}_{\text{e}}) \cdot m_i(\mathbf{r}) \, d\mathbf{r}
\end{cases}
$$

where ϕ_{exc} is the excitation photon flux, Δt the acquisition time, $h_{\text{exc}}(\mathbf{r}_{\text{e}})$ the probability distribution in space of the excitation photons, σ_{abs} the absorption cross-section, Φ_{fl} the fluorescence quantum yield, η_{det} the detection efficiency of the detector, $h_{\text{em}}(\mathbf{r})$ the probability distribution in space of the emitted photons and $m_i(\mathbf{r})$ the binary mask of the i-th detecting element. We insert this in Eq. S.4 and obtain

$$
\begin{aligned}
s_i(\mathbf{r}_{\text{e}}) &= \phi_{\text{exc}} \cdot \Delta t \cdot h_{\text{exc}}(\mathbf{r}_{\text{e}}) \cdot \sigma_{\text{abs}} \cdot \Phi_{\text{fl}} \cdot \eta_{\text{det}} \cdot \int_{\mathbb{R}^3} h_{\text{em}}(\mathbf{r} - \mathbf{r}_{\text{e}}) \cdot m_i(\mathbf{r}) \, d\mathbf{r} = \\
&= \Big(\phi_{\text{exc}} \, \Delta t \, \sigma_{\text{abs}} \, \Phi_{\text{fl}} \, \eta_{\text{det}}\Big) \cdot h_{\text{exc}}(\mathbf{r}_{\text{e}}) \Big(h_{\text{em}} \star m_i\Big)(\mathbf{r}_{\text{e}}) = \\
&= C \cdot h_{\text{exc}}(\mathbf{r}_{\text{e}}) \Big(h_{\text{em}} \star m_i\Big)(\mathbf{r}_{\text{e}}) = \\
&= C \cdot h_i(\mathbf{r}_{\text{e}})
\end{aligned}
\tag{S.5}
$$

where we group all the space independent parameters and the photophysics of the particle in a new constant C. The $\star$ denotes the cross-correlation operation. The spatial variation of the signal countings is described by the distributions $h_i(\mathbf{r}_{\text{e}}) = h_{\text{exc}}(\mathbf{r}_{\text{e}}) \cdot (h_{\text{em}} \star m_i)(\mathbf{r}_{\text{e}})$, which we identify as the single-detector point spread functions (PSFs) of our laser scanning microscope [3, 4]. Each single-detector PSF accounts for the combined effects of the illumination shape and the relative position of each detecting element. Consequently, these maps do not merely result from simple geometrical shifts. In fact, since the excitation is diffraction-limited (Supplementary Fig. 1a,b) and coaligned with the central element of the detector, the peripheral elements are disadvantaged in collecting photons. Despite featuring the same binary mask shape $m_i(\mathbf{r})$, they operate in regions with significantly lower excitation probability $P(\text{excitation})$ (Supplementary Fig. 1c,d).

By merging Eqs. S.2, S.3 and S.5, we finally calculate the multinomial parameters in absence of background

$$
\begin{aligned}
p_k^{(0)}(\mathbf{r}_\mathrm{e}) &= \frac{s_k(\mathbf{r}_\mathrm{e})}{\sum_{i=1}^{K} s_i(\mathbf{r}_\mathrm{e})} = \\
&= \frac{C \cdot h_\mathrm{exc}(\mathbf{r}_\mathrm{e}) \Big(h_\mathrm{em} \star m_k \Big)(\mathbf{r}_\mathrm{e})}{\sum_{i=1}^{K} C \cdot h_\mathrm{exc}(\mathbf{r}_\mathrm{e}) \Big(h_\mathrm{em} \star m_i \Big)(\mathbf{r}_\mathrm{e})} = \\
&= \frac{C \cdot h_\mathrm{exc}(\mathbf{r}_\mathrm{e}) \Big(h_\mathrm{em} \star m_k \Big)(\mathbf{r}_\mathrm{e})}{C \cdot h_\mathrm{exc}(\mathbf{r}_\mathrm{e}) \cdot \sum_{i=1}^{K} \Big(h_\mathrm{em} \star m_i \Big)(\mathbf{r}_\mathrm{e})} = \\
&= \frac{\Big(h_\mathrm{em} \star m_k \Big)(\mathbf{r}_\mathrm{e})}{\sum_{i=1}^{K} \Big(h_\mathrm{em} \star m_i \Big)(\mathbf{r}_\mathrm{e})}
\end{aligned}
\tag{S.6}
$$

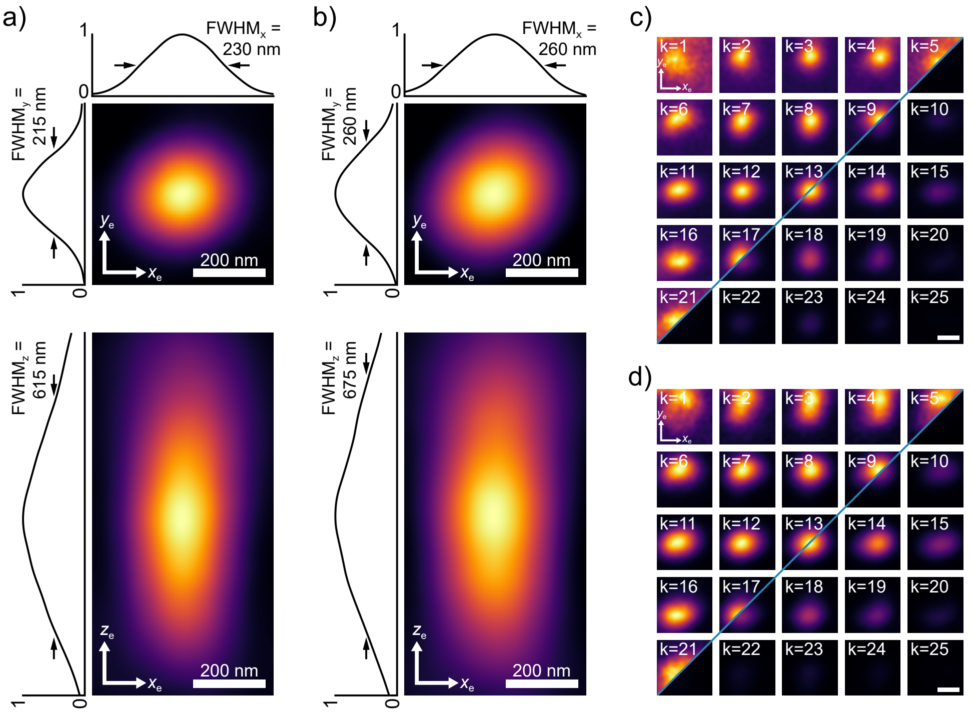

Supplementary Figure 1 Experimental characterization of the single-detector point spread function. **a-b)** Measured 3D excitation PSFs $h_\mathrm{exc}(\mathbf{r}_\mathrm{e})$ at $\lambda_\mathrm{exc} = 488\,\mathrm{nm}$ (**a**) and at $\lambda_\mathrm{exc} = 561\,\mathrm{nm}$ (**b**). Each experiment utilizes a 20 nm fluorescent bead at the appropriate wavelength for probing the excitation. **c-d)** Measured single-detector PSFs $h_k(\mathbf{r}_\mathrm{e})$ at $\lambda_\mathrm{exc} = 488\,\mathrm{nm}$ (**c**) and at $\lambda_\mathrm{exc} = 561\,\mathrm{nm}$ (**d**). The PSFs are arranged in 5×5 grid to help identify the corresponding SPAD array detector element. Upper diagonal images are normalized to themselves, while lower diagonal ones are normalized to the global maximum of the whole set. Each experiment utilizes a 20 nm fluorescent bead at the appropriate wavelength to probe the PSF. Scale bar = 200 nm.

Here it is relevant to note how the excitation cancels out of the equation and the distribution of the photon countings $\mathbf{n}$ solely depends on the detection geometry.

A more sophisticated model incorporates background. By dividing the photon counts into contributions from signal and background $n_i = n_i^s + n_i^{bkg}$, and assuming both signal and background follow Poisson distributions $n_i^s \sim \text{Poisson}(s_i)$ and $n_i^{bkg} \sim \text{Poisson}(b_i)$, we can extend Eq. S.3 as follows:

$$\lambda_i(\mathbf{r}_\mathrm{e}) = s_i(\mathbf{r}_\mathrm{e}) + b_i \tag{S.7}$$

and apply the usual definition of the signal-to-noise ratio (SBR):

$$\mathrm{SBR}(\mathbf{r}_\mathrm{e}) = \frac{\sum_{i=1}^{K} s_i(\mathbf{r}_\mathrm{e})}{\sum_{i=1}^{K} b_i} \tag{S.8}$$

We define the scalar parameter $\mathrm{SBR_p} \equiv \mathrm{SBR}(\mathbf{0})$ as the peak value of the SBR calculated for a particle perfectly in focus (i.e. in the center of the excitation volume $\mathbf{r}_\mathrm{e} = \mathbf{0}$). We obtain

$$\begin{aligned}
\mathrm{SBR}(\mathbf{r}_\mathrm{e}) &= \frac{\sum_{i=1}^{K} C \cdot h_i(\mathbf{r}_\mathrm{e})}{\sum_{i=1}^{K} b_i} = \\
&= \frac{C \cdot \sum_{i=1}^{K} h_i(\mathbf{0})}{\sum_{i=1}^{K} b_i} \cdot \frac{\sum_{i=1}^{K} h_i(\mathbf{r}_\mathrm{e})}{\sum_{i=1}^{K} h_i(\mathbf{0})} = \\
&= \mathrm{SBR}(\mathbf{0}) \cdot \frac{\sum_{i=1}^{K} h_i(\mathbf{r}_\mathrm{e})}{\sum_{i=1}^{K} h_i(\mathbf{0})} = \\
&= \mathrm{SBR_p} \cdot \frac{\sum_{i=1}^{K} h_i(\mathbf{r}_\mathrm{e})}{\sum_{i=1}^{K} h_i(\mathbf{0})}
\end{aligned} \tag{S.9}$$

The new multinomial parameters are:

$$\begin{aligned}
p_k(\mathbf{r}_\mathrm{e}) &== \frac{s_k(\mathbf{r}_\mathrm{e}) + b_k}{\sum_{i=1}^{K} [s_i(\mathbf{r}_\mathrm{e}) + b_i]} = \\
&= \frac{s_k(\mathbf{r}_\mathrm{e}) + b_k}{\sum_{i=1}^{K} s_i(\mathbf{r}_\mathrm{e}) + \sum_{i=1}^{K} s_i(\mathbf{r}_\mathrm{e})/\mathrm{SBR}(\mathbf{r}_\mathrm{e})} = \\
&= \frac{s_k(\mathbf{r}_\mathrm{e})}{(1 + 1/\mathrm{SBR}(\mathbf{r}_\mathrm{e})) \cdot \sum_{i=1}^{K} s_i(\mathbf{r}_\mathrm{e})} + \frac{b_k}{(1 + 1/\mathrm{SBR}(\mathbf{r}_\mathrm{e})) \cdot \sum_{i=1}^{K} s_i(\mathbf{r}_\mathrm{e})} = \\
&= \frac{\mathrm{SBR}(\mathbf{r}_\mathrm{e})}{\mathrm{SBR}(\mathbf{r}_\mathrm{e}) + 1} \cdot \frac{s_k(\mathbf{r}_\mathrm{e})}{\sum_{i=1}^{K} s_i(\mathbf{r}_\mathrm{e})} + \frac{1}{\mathrm{SBR}(\mathbf{r}_\mathrm{e}) + 1} \cdot \frac{b_k}{\sum_{i=1}^{K} b_i}
\end{aligned}$$

We can finally substitute the ratio between the expected values s_i with the multinomial parameters in absence of background of Eq. S.6, obtaining

$$p_k(\mathbf{r}_\mathrm{e}) = \frac{\mathrm{SBR}(\mathbf{r}_\mathrm{e})}{\mathrm{SBR}(\mathbf{r}_\mathrm{e})+1} \cdot p_k^{(0)}(\mathbf{r}_\mathrm{e}) + \frac{1}{\mathrm{SBR}(\mathbf{r}_\mathrm{e})+1} \cdot \frac{b_k}{\sum_{i=1}^{K} b_i} \tag{S.10}$$

We then use Eq. S.1 to calculate the Fisher information matrix. For the 2D case $\mathbf{r}_\mathrm{e} = (x_\mathrm{e}, y_\mathrm{e})$ we calculate:

$$\begin{aligned}
\mathbf{F}(\mathbf{r}_\mathrm{e}) &= -\mathbb{E}\left(\begin{bmatrix} \frac{\partial^2 \ln[\mathcal{L}(\mathbf{r}_\mathrm{e}|\mathbf{n})]}{\partial x^2} & \frac{\partial^2 \ln[\mathcal{L}(\mathbf{r}_\mathrm{e}|\mathbf{n})]}{\partial x \partial y} \\ \frac{\partial^2 \ln[\mathcal{L}(\mathbf{r}_\mathrm{e}|\mathbf{n})]}{\partial x \partial y} & \frac{\partial^2 \ln[\mathcal{L}(\mathbf{r}_\mathrm{e}|\mathbf{n})]}{\partial y^2} \end{bmatrix}\right) = \\
&= N \cdot \sum_{k=1}^{K} \frac{1}{p_k} \cdot \begin{bmatrix} \left(\frac{\partial p_k(\mathbf{r}_\mathrm{e})}{\partial x}\right)^2 & \frac{\partial p_k(\mathbf{r}_\mathrm{e})}{\partial x}\frac{\partial p_k(\mathbf{r}_\mathrm{e})}{\partial y} \\ \frac{\partial p_k(\mathbf{r}_\mathrm{e})}{\partial y}\frac{\partial p_k(\mathbf{r}_\mathrm{e})}{\partial x} & \left(\frac{\partial p_k(\mathbf{r}_\mathrm{e})}{\partial y}\right)^2 \end{bmatrix} = \\
&= N \cdot \mathbf{F}_\mathrm{n}(\mathbf{r}_\mathrm{e})
\end{aligned} \tag{S.11}$$

where we separate the dependency on space by defining the normalized Fisher matrix $\mathbf{F}_\mathrm{n}(\mathbf{r}_\mathrm{e})$. Finally, we obtain the CRB 2D maps $\sigma_\mathrm{CRB}(x_\mathrm{e}, y_\mathrm{e}) = \left[\sigma_x(x_\mathrm{e}, y_\mathrm{e}), \sigma_y(x_\mathrm{e}, y_\mathrm{e})\right]$ as

$$\begin{aligned}
\sigma_\mathrm{CRB}(x_\mathrm{e}, y_\mathrm{e}) &= \sqrt{\mathrm{Eig}\Big(\mathbf{F}^{-1}(\mathbf{r}_\mathrm{e})\Big)} = \\
&= \sqrt{\mathrm{Eig}\Big(\big(N \cdot \mathbf{F}_\mathrm{n}(\mathbf{r}_\mathrm{e})\big)^{-1}\Big)} = \\
&= \sqrt{\mathrm{Eig}\Big(\frac{1}{N} \cdot \mathbf{F}_\mathrm{n}^{-1}(\mathbf{r}_\mathrm{e})\Big)} = \\
&= \frac{1}{\sqrt{N}} \cdot \sqrt{\mathrm{Eig}\Big(\mathbf{F}_\mathrm{n}^{-1}(\mathbf{r}_\mathrm{e})\Big)}
\end{aligned} \tag{S.12}$$

with $\mathrm{Eig}(\mathbf{A})$ denoting the set of eigenvalues of the matrix $\mathbf{A}$. The shape of the CRB maps clearly depends on the normalized Fisher matrix $\mathbf{F}_\mathrm{n}(\mathbf{r}_\mathrm{e})$ (i.e. on the multinomial parameters $p_\mathrm{k}(\mathbf{r}_\mathrm{e})$). However, it's important to highlight how the absolute values of the uncertainties scale with $\sigma_\mathrm{CRB}(x_\mathrm{e}, y_\mathrm{e}) \propto 1/\sqrt{N}$.

In this regard, it is worth discussing the value of N we should input the formula. In wide-field camera-based methods, each sample position receives uniform illumination and its emission is equally collected, making the interpretation of N straightforward. However, in our technique we need to be aware we make use of a focused beam (Eq. S.5). In fact, given the same set of multinomial parameters p_k (Supplementary Fig. 2a), the CRB calculation can be performed in two alternative configurations by either fixing the number of detected photons N or make it variable depending on the excitation level at each position.

The first method suits single-molecule localization experiments, where N represents the available photon budget, collected irrespective of particle position. However, it's crucial to note that depending on the emitter's position $\mathbf{r}_e$, this may necessitate a significant increase in acquisition time. Furthermore, with the inclusion of background, the SBR remains spatially dependent (Eq. S.9). This results in peripheral positions generating fewer signal photons, which causes a deterioration of the localization precision (Supplementary Fig. 2b).

In the second scenario where the localizations are performed at a fixed acquisition time, such as in tracking, it is evident that the total number of detected countings N will be different depending on the position $\mathbf{r}_e$ (as well as the SBR). By defining the scalar parameter N_p as the peak number of detected photons for a particle perfectly in focus ($\mathbf{r}_e = \mathbf{0}$) and using Eq. S.9, we can derive $N(\mathbf{r}_e)$ including the noise:

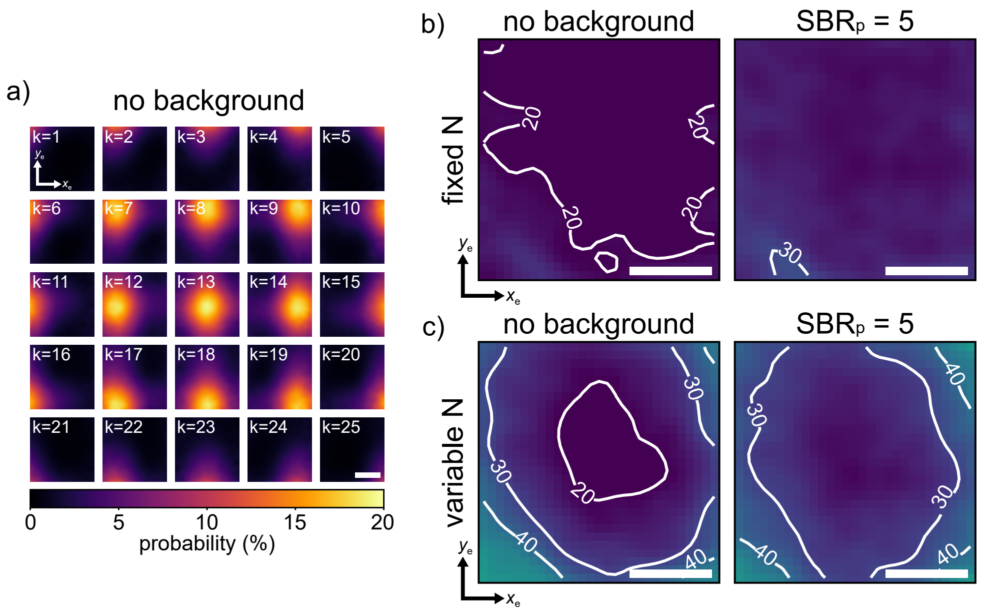

Supplementary Figure 2 Alternative benchmarking of the minimum localization uncertainty with an experimental point spread function. **a)** Experimental multinomial parameters $p_k^{(0)}$ in absence of background at $\lambda_{\mathrm{exc}} = 561\,\mathrm{nm}$. The maps are obtained applying Eq. S.6 with the experimental PSFs of Supplementary Fig. 1d. Scale bar = 200 nm. **b)** Lateral planar map $\sigma = \sqrt{\sigma_x^2 + \sigma_y^2}$ obtained with a fixed amount of detected photons N and the multinomial parameters in **a**. When the background is included, the multinomial parameters are modified according to Eq. S.10 and the value of the SBR depends on the emitter position as described by Eq. S.9. Scale bar = 100 nm. **c)** Lateral planar map $\sigma = \sqrt{\sigma_x^2 + \sigma_y^2}$ obtained with a variable amount of detected photons $N = N(\mathbf{r}_e)$ (Eq. S.13) and the multinomial parameters in **a**. When the background is included, the multinomial parameters are modified according to Eq. S.10 and the value of the SBR depends on the emitter position as described by Eq. S.9. Scale bar = 100 nm.

$$
\begin{aligned}
N(\mathbf{r}_\mathrm{e}) &= \sum_{i=1}^{K} \lambda_i(\mathbf{r}_\mathrm{e}) = \sum_{i=1}^{K} \big(s_i(\mathbf{r}_\mathrm{e}) + b_i\big) = \\
&= \sum_{i=1}^{K} \big(s_i(\mathbf{0}) + b_i\big) \cdot \frac{\sum_{i=1}^{K} \big(s_i(\mathbf{r}_\mathrm{e}) + b_i\big)}{\sum_{i=1}^{K} \big(s_i(\mathbf{0}) + b_i\big)} = \\
&= N_\mathrm{p} \cdot \frac{\sum_{i=1}^{K} \big(s_i(\mathbf{r}_\mathrm{e}) + b_i\big)}{\sum_{i=1}^{K} \big(s_i(\mathbf{0}) + b_i\big)} = \\
&= N_\mathrm{p} \cdot \frac{\sum_{i=1}^{K} s_i(\mathbf{r}_\mathrm{e}) + \sum_{i=1}^{K} s_i(\mathbf{r}_\mathrm{e})/\mathrm{SBR}(\mathbf{r}_\mathrm{e})}{\sum_{i=1}^{K} s_i(\mathbf{0}) + \sum_{i=1}^{K} s_i(\mathbf{r}_\mathrm{e})/\mathrm{SBR}(\mathbf{r}_\mathrm{e})} = \\
&= N_\mathrm{p} \cdot \frac{\big(\mathrm{SBR}(\mathbf{r}_\mathrm{e}) + 1\big) \cdot \sum_{i=1}^{K} s_i(\mathbf{r}_\mathrm{e})}{\mathrm{SBR}(\mathbf{r}_\mathrm{e}) \cdot \sum_{i=1}^{K} s_i(\mathbf{0}) + \sum_{i=1}^{K} s_i(\mathbf{r}_\mathrm{e})} = \\
&= N_\mathrm{p} \cdot \frac{\big(\mathrm{SBR}(\mathbf{r}_\mathrm{e}) + 1\big) \cdot \sum_{i=1}^{K} h_i(\mathbf{r}_\mathrm{e})}{\mathrm{SBR}(\mathbf{r}_\mathrm{e}) \cdot \sum_{i=1}^{K} h_i(\mathbf{0}) + \sum_{i=1}^{K} h_i(\mathbf{r}_\mathrm{e})} = \\
&= N_\mathrm{p} \cdot \frac{\big(\mathrm{SBR}(\mathbf{r}_\mathrm{e}) + 1\big) \cdot \sum_{i=1}^{K} h_i(\mathbf{r}_\mathrm{e})}{\mathrm{SBR}_\mathrm{p} \cdot \sum_{i=1}^{K} h_i(\mathbf{r}_\mathrm{e}) + \sum_{i=1}^{K} h_i(\mathbf{r}_\mathrm{e})} = \\
&= N_\mathrm{p} \cdot \frac{\mathrm{SBR}(\mathbf{r}_\mathrm{e}) + 1}{\mathrm{SBR}_\mathrm{p} + 1} = \\
&= N_\mathrm{p} \cdot \left[\frac{\mathrm{SBR}_\mathrm{p}}{\mathrm{SBR}_\mathrm{p} + 1} \cdot \frac{\sum_{i=1}^{K} h_i(\mathbf{r}_\mathrm{e})}{\sum_{i=1}^{K} h_i(\mathbf{0})} + \frac{1}{\mathrm{SBR}_\mathrm{p} + 1}\right]
\end{aligned}
\tag{S.13}
$$

As pointed out in the main text, the parameters SBR_p and N_p contains all the necessary information for describing the experimental condition, alongside with the knowledge of the system single-detector PSFs $h_k(\mathbf{r}_\mathrm{e})$. Notably, the uncertainties obtained in the tracking scenario are coherently worse than the ones obtained with a fixed photon budget. In particular, the performance rapidly degrades when the distance from the focal position increases (Supplementary Fig. 2c).

SI Note 2 Localization estimators characterization

Maximum likelihood estimator

The maximum likelihood estimator (MLE) calculates the position of the fluorophore by maximizing the likelihood function already described in Eq. S.1:

$$\hat{\mathbf{r}}_{\mathrm{e}} = \operatorname{argmax}\left(\mathcal{L}(\mathbf{r}_{\mathrm{e}}|\mathbf{n})\right) \tag{S.14}$$

Coherently with what already discussed in the derivation of the CRB, the estimation relies on the usage of a proper model of the PSF as well as the characterization of the noise represented by the parameter $\mathrm{SBR_p}$. The MLE experimentally provides an unbiased and linear localization in all directions (Supplementary Fig. 3).

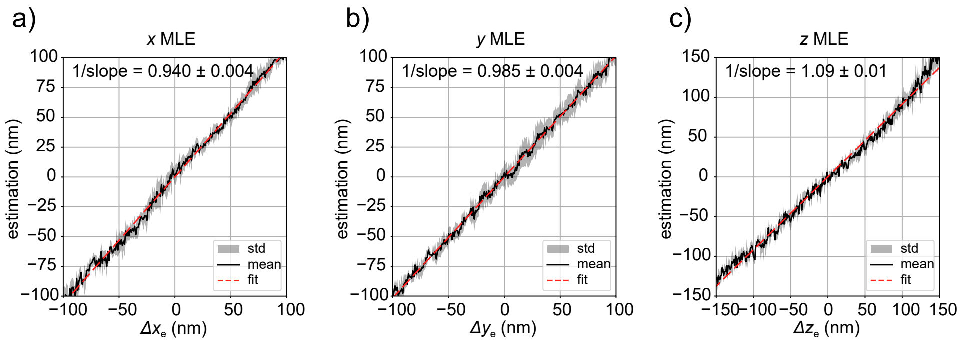

Supplementary Figure 3 Experimental characterization of the maximum likelihood estimator in the three spatial dimensions. a-c) Calibration curves obtained by moving with the piezoelectric stage a 40 nm fluorescent bead ($\lambda_{\mathrm{exc}} = 561$ nm) several times for each direction and localizing its position with the MLE ($\mathrm{SBR_p} = 50$). The shaded area is the standard deviation.

Fast estimators

The high amount of calculations necessary to perform the estimation with the MLE makes its implementation in real-time unfeasible. We consequently define here a set of faster estimators, which allows us to gain computational speed at the expense of precision and/or accuracy.

The information about any lateral displacements is contained in the position of the intensity distribution in the microimage. Therefore, we can use the centroid estimator to obtain a fast lateral localization of the particle. Given a microimage $I(i,j) \equiv I(i,j \mid \mathbf{r}_{\mathrm{e}})$ and proper calibration factors k_{x} and k_{y} we define:

$$
\hat{x}_{\mathrm{e}} = k_{\mathrm{x}} \cdot \frac{\sum\limits_{i=-2}^{2} \sum\limits_{j=-2}^{2} I(i,j) \cdot i}{\sum\limits_{i=-2}^{2} \sum\limits_{j=-2}^{2} I(i,j)}
\qquad
\hat{y}_{\mathrm{e}} = k_{\mathrm{y}} \cdot \frac{\sum\limits_{j=-2}^{2} \sum\limits_{j=-2}^{2} I(i,j) \cdot j}{\sum\limits_{i=-2}^{2} \sum\limits_{j=-2}^{2} I(i,j)} \tag{S.15}
$$

The centroid estimator correctly provides the position of the emitter as long as the intensity distribution in the microimage $I(i, j)$ is centrosymmetric with respect to the position to be estimated. This hypothesis holds in the absence of noise and when the emission is fully contained in the detector. When the position approaches the edge of the static field-of-view (sFoV) or noise is added, one or both of the requirements are not met and the performance is affected.

We use a simulated dataset to show the combination of the two parameters (Supplementary Fig. 4). The first deviation from the ideal estimation happens because of cropping: the limited sFoV fails to image properly the emission PSF and the resulting microimage has a centroid shifted towards the center of the coordinate system (Supplementary Fig. 4a). The effect of breaking the central symmetry is then worsened when noise is added (Supplementary Fig. 4b). The recorded microimage is the sum of the two independent patterns deriving from the signal and the background noise $I(i, j) = I_{\mathrm{s}}(i, j) + I_{\mathrm{bkg}}(i, j)$. Each provides its own contribution to the centroid:

$$
\hat{x}_{\mathrm{e}} = k_{\mathrm{x}} \cdot \frac{\sum\limits_{i=-2}^{2} \sum\limits_{j=-2}^{2} [I_{\mathrm{s}}(i,j) + I_{\mathrm{bkg}}(i,j)] \cdot i}{\sum\limits_{i=-2}^{2} \sum\limits_{j=-2}^{2} [I_{\mathrm{s}}(i,j) + I_{\mathrm{bkg}}(i,j)]} =
$$

$$
= k_{\mathrm{x}} \cdot \frac{\sum\limits_{i=-2}^{2} \sum\limits_{j=-2}^{2} I_{\mathrm{s}}(i,j) \cdot i}{\sum\limits_{i=-2}^{2} \sum\limits_{j=-2}^{2} [I_{\mathrm{s}}(i,j) + I_{\mathrm{bkg}}(i,j)]} + k_{\mathrm{x}} \cdot \frac{\sum\limits_{i=-2}^{2} \sum\limits_{j=-2}^{2} I_{\mathrm{bkg}}(i,j) \cdot i}{\sum\limits_{i=-2}^{2} \sum\limits_{j=-2}^{2} [I_{\mathrm{s}}(i,j) + I_{\mathrm{bkg}}(i,j)]}
$$

We can rewrite the definition of the SBR in Eq. S.8 using the microimage notation

$$
\mathrm{SBR} = \frac{\sum_{i=-2}^{2} \sum_{j=-2}^{2} I_{\mathrm{s}}(i,j)}{\sum_{i=-2}^{2} \sum_{j=-2}^{2} I_{\mathrm{bkg}}(i,j)} \tag{S.16}
$$

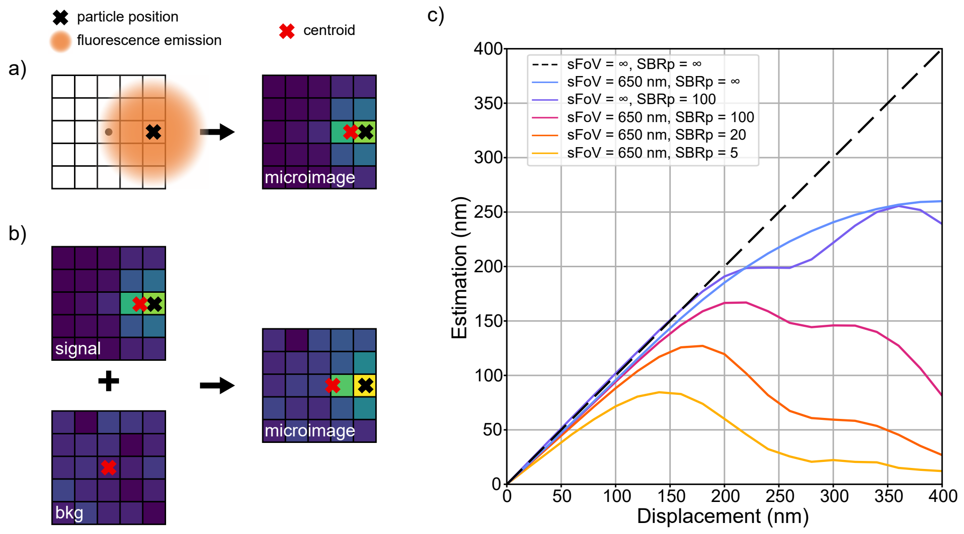

Supplementary Figure 4 Simulated linearity of the centroid estimator as a function of the static field-of-view and the signal-to-noise ratio. **a)** When the limited physical size of the detector is taken into account, the shifted emission PSF (orange spot) is cropped and the resulting microimage is not centrosymmetric anymore. Consequently, there is a difference between the estimated position $\hat{\mathbf{r}}_e$ (red cross) and the actual one (black cross), with the former dragged towards the center of the sFoV. **b)** The presence of background noise ($\mathrm{SBR_p} = 50$) effectively introduces another source of photons that adds up to the signal. The estimation of **a** is further modified by the centroid of this extra pattern and shows a bigger deviation from the ideal case. **c)** Simulated centroid estimation of a lateral shift for different combinations of sFoV size and SBR level.

where we drop the explicit dependency over the emitter position $\mathrm{SBR} \equiv \mathrm{SBR}(\mathbf{r}_e)$ for improving the readability. Defining $\hat{x}_s$ and $\hat{x}_{bkg}$ the centroid estimates of the signal and background patterns, we obtain:

$$\hat{x}_e = \frac{\mathrm{SBR}}{\mathrm{SBR}+1} \cdot \hat{x}_s + \frac{1}{\mathrm{SBR}+1} \cdot \hat{x}_{bkg} \tag{S.17}$$

A similar formula can be derived for $\hat{y}_e$. Notably, the central symmetry of the emission PSF is preserved when the particle is shifted in the axial direction, making the lateral localization with the centroid independent of the axial position.

The axial information, on the contrary, is encoded in the shape of the astigmatic emission PSF [5]. To leverage this feature, we define what we call the normalized difference estimator:

$$\hat{z}_{\mathrm{e}} = k_{\mathrm{z}} \cdot \frac{\sum\limits_{i=-2}^{2} I(i,0) - \sum\limits_{j=-2}^{2} I(0,j)}{\sum\limits_{i=-2}^{2}\sum\limits_{j=-2}^{2} I(i,j)} = \\ = k_{\mathrm{z}} \cdot \frac{s_{\mathrm{v}}(0) - s_{\mathrm{h}}(0)}{\sum\limits_{i=-2}^{2}\sum\limits_{j=-2}^{2} I(i,j)} \tag{S.18}$$

where k_{z} is a calibration factor and $s_{\mathrm{v}}(j)$ and $s_{\mathrm{h}}(i)$ are the sums performed along the j-th column and i-th row.

Intuitively, the direction along which the PSF is more elongated is the one containing the biggest fraction of the intensity. Assuming the emission is centered at the coordinates $(i_{\mathrm{p}}, j_{\mathrm{p}})$ and is horizontally stretched, then the sum of the intensity values along its main horizontal axis $s_{\mathrm{h}}(i_{\mathrm{p}})$ is bigger than the sum along the main vertical axis $s_{\mathrm{v}}(j_{\mathrm{p}})$. Since this is a pure geometrical effect, the division by the overall intensity is necessary to normalize the difference. To gain computational speed, we decide to not estimate the center of the emission PSF and to arbitrarily perform the sums over the central row and central column of the detector ($s_{\mathrm{h}}(0)$ and $s_{\mathrm{v}}(0)$). As a consequence, the estimator is linear and unbiased only when the emission is centered in the microimage and bias arises with increasing lateral displacements. A shift in the y_{e} direction leads to a positive bias (Supplementary Fig. 5a) while a shift in the x_{e} direction leads to a negative bias (Supplementary Fig. 5b).

In theory, the convergence of the feedback loop is threatened by a biased axial estimation, but this negative outcome is, in practice, counterbalanced by two main effects. Firstly, the tracking procedure in the lateral direction keeps the sFoV locked on top of the particle and compensates for any lateral shift. Secondly, assuming the particle is in focus at the beginning of each estimation window, its intensity decreases while drifting laterally, according to the excitation PSF. Therefore the emission from peripheral positions contributes less to the microimage and the apparent position of the integrated intensity distribution is dragged towards the center of the detector.

In case the experiment requires it, it is nevertheless a good advice to make the axial estimation slower than the lateral one, improving axial accuracy at the cost of time resolution.

Despite all this possible sources of non-idealities, the centroid and normalized difference experimentally performs linearly in a region of hundreds of nanometers (Supplementary Fig. 6).

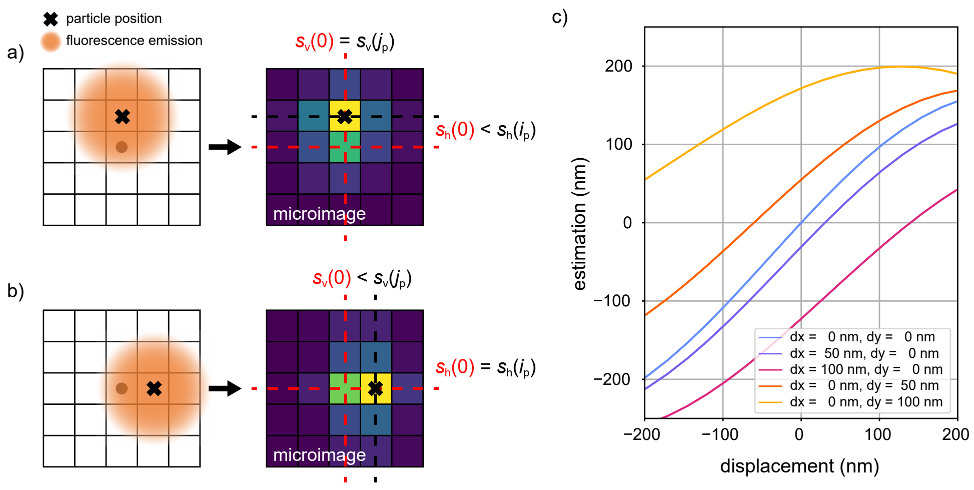

Supplementary Figure 5 Simulated bias and linearity of the normalized difference estimator as a function of the lateral displacement. a) When the emission is shifted in the vertical direction (in this case $dy = 100\,\mathrm{nm}$), the sum of the intensity in the central row $s_\mathrm{h}(0)$ (red) is smaller than the sum of the intensity along the main horizontal axis of the emission PSF $s_\mathrm{h}(i_\mathrm{p})$ (black). The estimation $\hat{z}_\mathrm{e} \propto s_\mathrm{v}(0) - s_\mathrm{h}(0)$ is therefore biased towards the positive axial direction. **b)** A shift in the horizontal direction ($dx = 100\,\mathrm{nm}$) causes an analog situation as depicted in **a**. The sum along the central column $s_\mathrm{v}(0)$ is smaller than the one of the main vertical axis $s_\mathrm{v}(j_\mathrm{p})$ and the estimation $\hat{z}_\mathrm{e}$ is consequently biased towards the negative axial direction. **c)** Simulated axial estimation curves of the normalized difference estimator with increasing shifts in both the lateral directions (dx and dy).

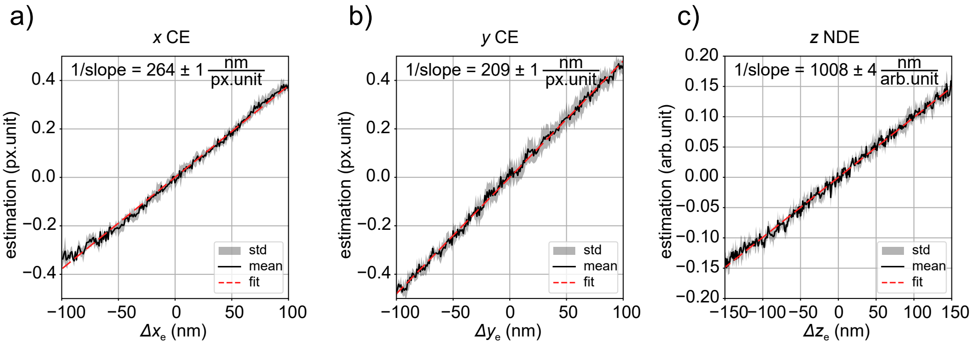

Supplementary Figure 6 Experimental characterization of the centroid and normalized differential estimators. a-c) The localization is performed laterally with the centroid and axially with the normalized difference estimators on the same dataset of Supplementary Fig. 3. The shaded area is the standard deviation.

SI Note 3 Estimation of the maximum measurable diffusion coefficient

A particle immersed in a fluid which is not subject to any driving force but the collisions with its surrounding molecules exhibits a peculiar random diffusion which is known as Brownian motion. According to Einstein formula, a Brownian trajectory $\mathbf{x}(t)$ is characterized by its diffusion coefficient D

$$\mathrm{MSD}(\delta t) \equiv \left\langle \left\| \mathbf{x}(t) - \mathbf{x}(t+\delta t) \right\|^2 \right\rangle_t = 2nD \cdot \delta t \tag{S.19}$$

where n is the number of dimensions of the trajectory.

Although many biological particles involved in diffusion processes deviate from the ideal case of free diffusion, the diffusion coefficient remains a valuable benchmark for comparing the tracking capabilities of different techniques. Calculating the maximum measurable diffusion coefficient requires therefore two characterizations: (1) a good

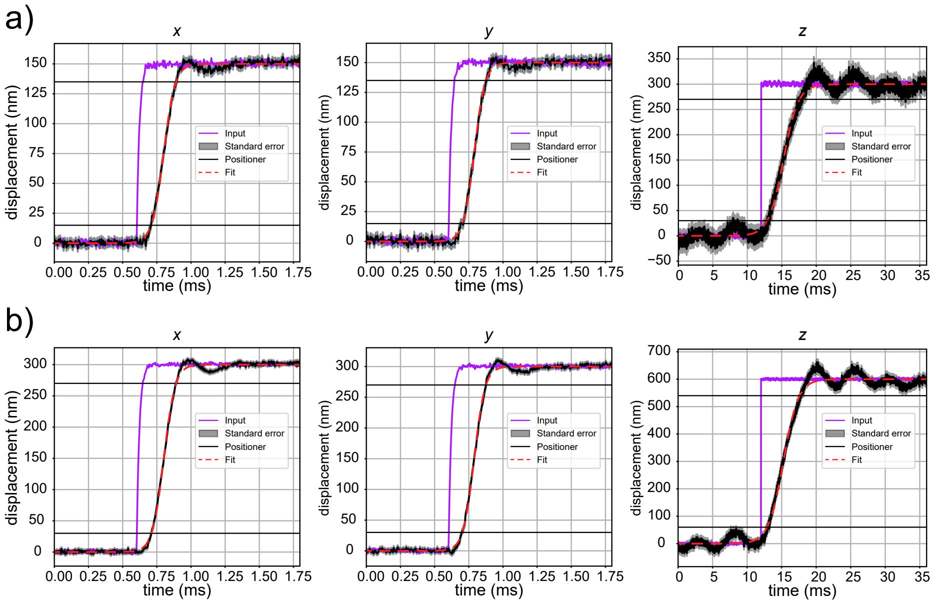

Supplementary Figure 7 Rise time of the positioners. **a)** Comparison between the input signal (violet) and the corresponding positioner response (black). All readings are taken from the positioner driver's sensor output port, except for the z-axis input signal which is taken directly from the FPGA. The displacement imposed in each direction matches with the half-widht of the OLV. The response is fitted with a logistic function (red) with the dashed lines marking the 10 % and 90 % levels from which the rise time t_r is calculated. The curves in x are the average of 898 cycles and yield $t_\mathrm{r}^\mathrm{x} = 185 \pm 2\,\mu\mathrm{s}$. The curves in y are the average of 897 cycles and yield $t_\mathrm{r}^\mathrm{y} = 174 \pm 2\,\mu\mathrm{s}$. The curves in z are the average of 597 cycles and yield $t_\mathrm{r}^\mathrm{z} = 4.71 \pm 0.03\,\mathrm{ms}$. The shaded area is the standard error of the mean. **b)** Same analysis as in **a** with the doubled target displacement. The curves in x are the average of 900 cycles and yield $t_\mathrm{r}^\mathrm{x} = 181 \pm 2\,\mu\mathrm{s}$. The curves in y are the average of 900 cycles and yield $t_\mathrm{r}^\mathrm{y} = 173 \pm 2\,\mu\mathrm{s}$. The curves in z are the average of 598 cycles and yield $t_\mathrm{r}^\mathrm{z} = 4.75 \pm 0.03\,\mathrm{ms}$. The shaded area is the standard error of the mean.

estimate of the farthest distance a particle can move while still being reliably detected and (2) the shortest possible time it takes for the particle to travel that distance.

We can figure out the first parameter based on the dimension of the optimal localization volume (OLV). If a particle is in the center of the sFoV at the beginning of the estimation window, it can move at most 150 nm laterally and 300 nm axially. The second parameter on the contrary depends on the speed at which the positioners can re-center the scanning position $\mathbf{r}_\mathrm{s}$. A useful benchmark is the rise time t_r which is defined as the time it takes a signal to rise from 10 % to 90 % of the target value. We measured the rise time relative to the previous displacements as $t_\mathrm{r}^\mathrm{x} = 185\,\mu\mathrm{s}$, $t_\mathrm{r}^\mathrm{y} = 174\,\mu\mathrm{s}$ and $t_\mathrm{r}^\mathrm{z} = 4.71\,\mathrm{ms}$ (Supplementary Fig. 7a).

Using Equation S.19, we estimate the maximum measurable diffusion coefficients: $D_\mathrm{max}^\mathrm{x} \approx 61\,\mu\mathrm{m}^2/\mathrm{s}$, $D_\mathrm{max}^\mathrm{y} \approx 65\,\mu\mathrm{m}^2/\mathrm{s}$ and $D_\mathrm{max}^\mathrm{z} \approx 9.6\,\mu\mathrm{m}^2/\mathrm{s}$. For this analysis, the z-axis performance, with its lower D_max, limits the overall capability. To improve the maximum measurable diffusion coefficient, two main solutions exist: increasing the positioner speed or expanding the OLV. Modifying the excitation volume or sFoV would alter the experimental setup, while the re-centering speed can potentially be boosted without changing the positioners themselves. This could be achieved through optimized input signal design. For example, doubling the positioner displacement doesn't change the rise time (Supplementary Fig. 7b), suggesting possibilities for an overshoot scheme exceeding the current implementation's speed.

SI Note 4 Measurement of the hydrodynamic radius using single-particle tracking

To further assess the reliability of our single-particle tracking (SPT) technique, we propose measuring the hydrodynamic radius of fluorescent beads of known dimension from their free diffusion 3D trajectories. According to the Stokes–Einstein–Sutherland equation, the Brownian motion of a spherical particle diffusing in a fluid of viscosity η is characterized by:

$$D = \frac{k_B T}{6\pi\eta r_{hyd}} \tag{S.20}$$

where D is the diffusion coefficient, k_B is Boltzmann constant, T is the temperature and r_{hyd} is the hydrodynamic radius.

In our experiment, we systematically vary the viscosity by preparing four water-glycerol mixtures with different volumetric ratios of glycerol to total volume $R_{gly} = V_{gly}/V_{tot}$. Fluorescent beads (40 nm diameter) are added at a low concentration, ensuring an average of less than one bead within the sFoV at any time. For each mixture we acquire N_{tr} independent trajectories $\mathbf{r}^{(\mathbf{n})}(t) = \mathbf{r}_s^{(\mathbf{n})}(t)$, from which we calculate the overall averaged mean squared displacement (MSD) (see Methods). Each MSD curve (Supplementary Fig. 8) displays a behavior consistent with 3D Brownian motion

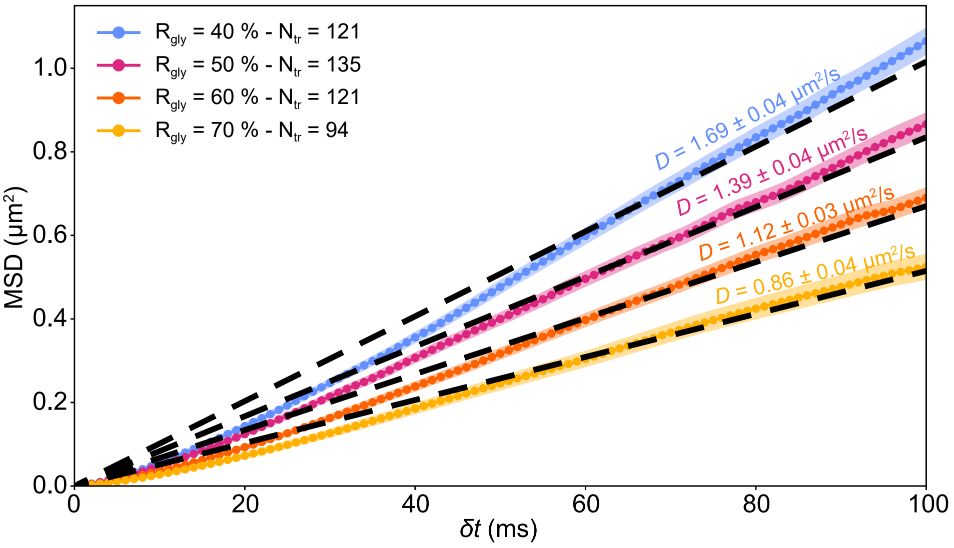

Supplementary Figure 8 3D tracking of free fluorescent beads in water-glycerol dilutions. MSD of 40 nm fluorescent beads ($\lambda_{exc} = 561$ nm) diffusing in different glycerol/water volumetric dilutions R_{gly}. Each curve is averaged over a number N_{tr} of independent trajectories obtained with real-time tracking at a fixed re-centering time $\Delta t_{rc}^{all} = 2.5$ ms. The shaded area is the standard error of the mean. Under the assumption of 3D Brownian motion, the MSD is fit with a linear model to extract the corresponding diffusion coefficient $D(R_{gly})$.

as described by Eq. S.19. This agreement justifies using these results in Eq. S.20 to estimate the hydrodynamic radius.

The viscosity of each solution is estimated using the method described by Volk et al. [6] and assuming a standard temperature of 293 K. The four measurements provide an average hydrodynamic radius of 16.0 ± 3.5 nm which corresponds to a bead diameter of 32 ± 7 nm. This value is compatible with the manufacturer's specification of 37 ± 6 nm.

The primary source of uncertainty in our estimation stems from the volumetric ratios R_{gly}.We conservatively assigned a relative error of $\epsilon_{\mathrm{r}} = 10\,\%$ to account for potential random errors and miscalibrations in measurements or equipment. For higher precision real-world applications it is desirable to aim for stricter control over R_{gly} or directly measure the medium's viscosity

SI Note 5 Statistical lifetime-based segmentation of the lysosome motion pattern

The correlation between the fluorescence lifetime and the motion state of lysosomes has potential implications for studying their diffusion behaviors. In fact, by analyzing the value of the fluorescence lifetime, it may be possible to segment and study the motion of lysosomes in a simple and effective way, without requiring the user to manually threshold the data (Methods). To test this hypothesis, we collect a set of 15 independent 4D trajectories $[x_s(t), y_s(t), z_s(t), \tau(t)]$.

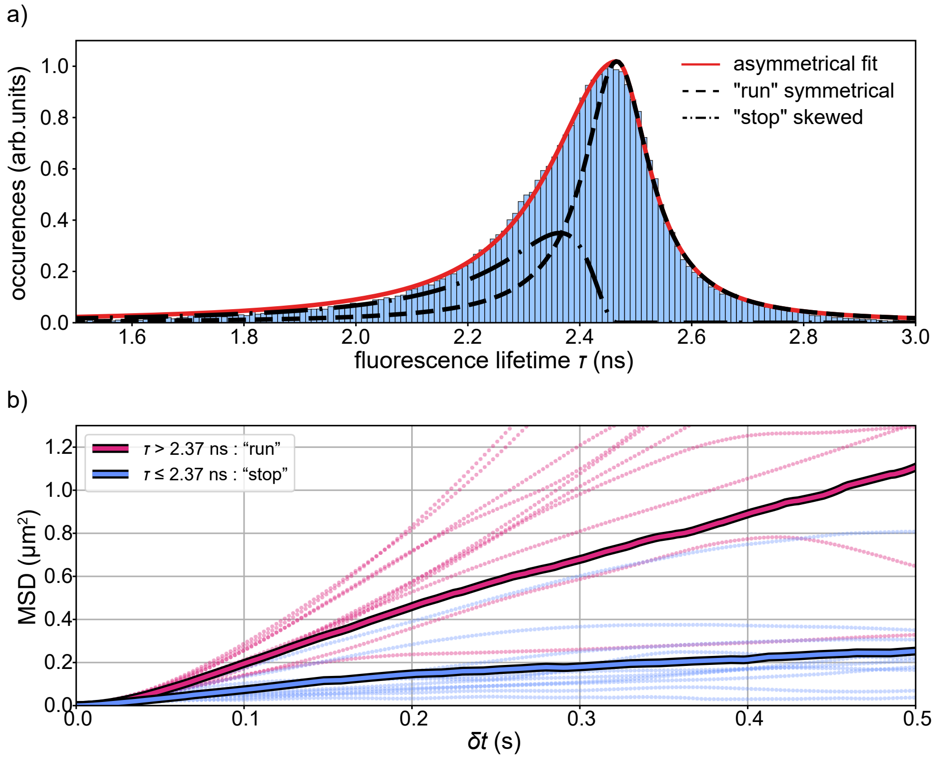

Supplementary Figure 9 Segmentation of the lysosome state by the fluorescence lifetime. **a)** Histogram of the fluorescence lifetime values measured in 15 independent RT-4D-SPT experiments on lysosomes. The shape is fitted with a custom asymmetrical Cauchy-Lorentz distribution for separating the contributions of the two motion states. The optimal segmentation threshold results in $\tau_{\text{th}} \approx 2.37\,\text{ns}$. **b)** Result of the segmentation by thresholding the fluorescence lifetime traces as described in **a**. The solid line curves are obtained by averaging the MSD of all the matching segments of all the 15 experiments (206 trajectory segments with $\tau > 2.37\,\text{ns}$ and 111 trajectory segments with $\tau \leq 2.37\,\text{ns}$). The dotted curves show the MSD of 10 randomly selected trajectory segments for each segmentation label. The re-centering is performed at a fixed dwell time $\Delta t_{\text{rc}}^{\text{all}} = 2.5\,\text{ms}$ and the lifetime is measured every $\Delta t_{\tau} = 10\,\text{ms}$.

The histogram of all the measured fluorescent lifetime values for all the trajectories reveals a negatively skewed distribution (Supplementary Fig. 9a). This is consistent with the decrease in lifetime during the "stop" state previously reported in the main text. To determine an optimal threshold value for the diffusion behavior, it is necessary to separate the two contributions. In the absence of a physical model that would allow us to derive a theoretical fitting function, we define a custom skewed curve based on the Cauchy-Lorentz distribution:

$$f(\tau; k, \mu, \gamma_\mathrm{r}, \gamma_\mathrm{l}) = \begin{cases} k \cdot \frac{\gamma_\mathrm{r}}{\gamma_\mathrm{r}^2 + (\tau-\mu)^2} & \text{if } \tau > \mu \\ \frac{k}{\gamma_\mathrm{r}} \cdot \frac{\gamma_\mathrm{l}^2}{\gamma_\mathrm{l}^2 + (\tau-\mu)^2} & \text{if } \tau \leq \mu \end{cases} \tag{S.21}$$

where μ is the mode of the distribution, γ_r and γ_l the right and left half width at half maximum and k is a scaling factor. The fitted curve agrees well with the experimental data, yielding the parameters $\mu = 2.466 \pm 0.001\,\mathrm{ns}$, $\gamma_\mathrm{r} = 0.071 \pm 0.001\,\mathrm{ns}$, and $\gamma_\mathrm{l} = 0.146 \pm 0.001\,\mathrm{ns}$.

Notably, the peak of f is in agreement with the expected fluorescence lifetime for the green fluorescent protein (GFP). If we assume that the "run" state generates a symmetrical Lorentzian distribution centered at μ, we can divide the custom function into two distinct contributions $f = f_\mathrm{run} + f_\mathrm{stop}$:

$$f_\mathrm{run}(\tau; k, \mu, \gamma_\mathrm{r}) = k \cdot \frac{\gamma_\mathrm{r}}{\gamma_\mathrm{r}^2 + (\tau-\mu)^2} \tag{S.22}$$

$$f_\mathrm{stop}(\tau; k, \mu, \gamma_\mathrm{r}, \gamma_\mathrm{l}) = \begin{cases} 0 & \text{if } \tau > \mu \\ \frac{k}{\gamma_\mathrm{r}} \cdot \frac{(\tau-\mu)^2 \cdot (\gamma_\mathrm{l}^2 - \gamma_\mathrm{r}^2)}{(\gamma_\mathrm{l}^2 + (\tau-\mu)^2) \cdot (\gamma_\mathrm{r}^2 + (\tau-\mu)^2)} & \text{if } \tau \leq \mu \end{cases} \tag{S.23}$$

Assuming f_run and f_stop are the probability distributions of measuring a certain τ when the lysosome is in a given motion state, we determine the optimal fluorescence lifetime threshold as the value at which both processes are equally likely $\tau_\mathrm{th} \approx 2.37\,\mathrm{ns}$. Trajectory points with a lifetime above τ_th are assigned the "run" state and the others the "stop" state. Each trajectory is therefore cut into several segments, each composed of subsequent points which are assigned the same motion state. To avoid spurious results we also discard segments shorter than 500 ms.

We expect the segments labeled as "run" state to display a faster movement compared to those labeled as "stop" state. To confirm this expectation, we compute the average MSD of the two populations (Supplementary Fig. 9b). The analysis reveals that the "run" state population indeed exhibits faster diffusion. Contrary to the expectation, the linearity of the curve suggests free diffusion. Nevertheless, we believe that the free diffusion motion is just an artifact caused by averaging all the "run" states of all the vesicles together. In fact, each MSD curve of single "run" state segments, exhibit a different non-Brownian diffusion, thus reflecting the variability of tasks performed by the lysosomes. On the other hand, the "stop" state curve shows a strong sub-diffusion behavior, which is an indication of confinement of the lysosomes.

SI Note 6 FPGA-based control module architecture and tracking algorithm

All the actions and computations necessary to control the microscope during the tracking experiments are performed in real-time by the dedicated control module. The control module is governed by a custom firmware developed in LabVIEW, which is integrated with a Graphical User Interface (GUI) on the PC (the "host"). This setup allows for dynamic user intervention and real-time data visualization. The hardware design consists in a chassis (NI PXIe-1071) housing a thunderbolt-based communication module (NI PXIe-8301) and two field-programmable gate array (FPGA)-based data-acquisition cards (the "targets"), operating in a master/slave configuration (Supplementary Fig. 10). Specifically, the chassis facilitates synchronization among all components through a shared clock running at 10 MHz, and enables internal communication between the two acquisition cards via eight independent trigger lines.

The digital card (NI PXIe-7822R) is an I/O digital data-acquisition (DAQ) and serves as the master of the system. It collects the single-photon pulses from the SPAD array and implements the real-time feedback loop. The digital input line from each SPAD element is checked at regular intervals of $\approx$1.97 ns = 1/508 MHz, ensuring lossless sampling of the read-out regardless of the selected detector hold-off time. The single-photon signals are then accumulated in the incremental photon-counting registers $\mathbf{n}$. To measure the photon timing, the time-to-digital converter (TDC) unit processes the photon-counting registers using the digital frequency domain (DFD) approach, enabling the calculation of a full histogram of the detection times $\mathbf{t}_\mathrm{d}$ every $\approx$5.7 µs = 1/176 kHz. Both $\mathbf{n}$ and $\mathbf{t}_\mathrm{d}$ are then fed to the logical unit, where they are processed at a cycle clock frequency of 20 MHz to trigger a real-time response.

Each new scanning position $\mathbf{r}_\mathrm{s}$ generated by the logical unit is immediately transmitted to the analogue card with a custom-written protocol that utilizes the eight internal trigger lines of the chassis. This protocol allows an entire message to be delivered in under 190.0 ± 2.5 ns.

The analogue card (NI PXIe-7856R) is an I/O analog and digital DAQ and acts as a slave. As such, it blindly converts control orders from external sources into voltage outputs. Specifically, the scanning position is sent from the digital card, while the piezoelectric stage position and the laser power are delivered from the host PC and can therefore be dinamically set by the user in the GUI. The speed of the electronic actuation is fixed by the digital-to-analog converter (DAC)'s minimum update time of 1 µs = 1/1 MHz, as specified by the seller.

The experimental raw data is sent to the host PC at two distinct timings. The photon-counting registers $\mathbf{n}$ and the scanning position $\mathbf{r}_\mathrm{s}$ are buffered at intervals of 2 µs = 1/500 kHz, while the detection time histograms $\mathbf{t}_\mathrm{d}$ are buffered at $k \cdot 10$ ns = k/100 MHz, where k is a tunable integer used to adjust the data rate and manage communication bandwidth.

The logical unit is the single most important block. It implements the feedback loop, and it is therefore responsible for effectively locking a single moving particle inside the detection volume. Before each experiment, the user sets the intended behavior of the logical unit, including the re-centering conditions for each spatial axis and

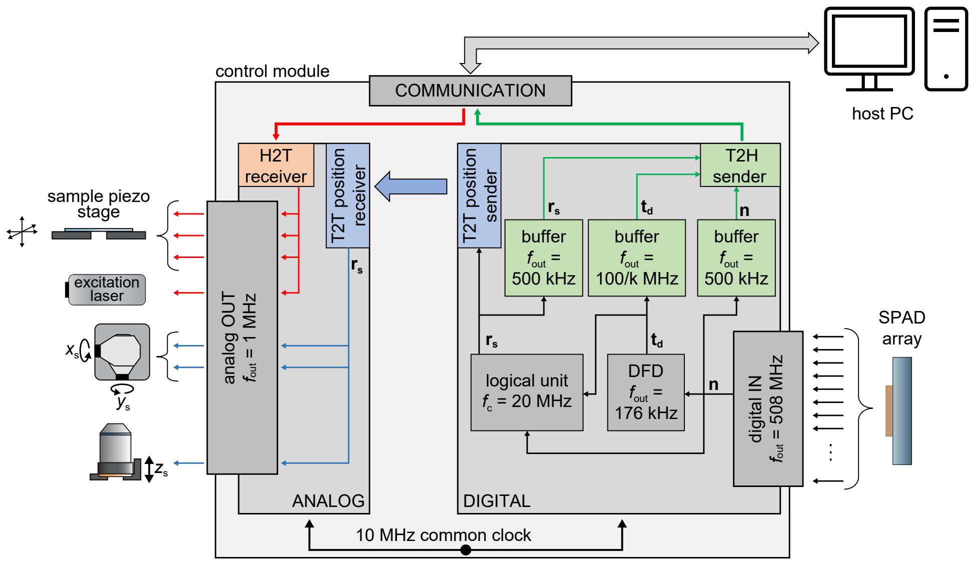

Supplementary Figure 10 Firmware of the control module for tracking. The chassis provides the two FPGA cards a common clock at 10 MHz from which all the other clocks are derived. Each functional block within the system operates at a specific output frequency f_{out}, indicating the rate at which its output becomes available. The logical unit, exceptionally, is characterized by its cycle frequency f_{c}, which denotes the clock rate at which the logical steps are executed (see Supplementary Fig.11). A valid output requires at least 4 cycles. In this schematic representation, green is used to mark the communication lines and blocks involved in transferring the experimental data from the digital target to the host PC (T2H). Conversely, red is used to indicate the path of the control commands relative to the position of the piezoelectric stage and the laser power from the host PC to the analog target (H2T) . The blue pathway represents the internal transfer of the scanning position from the digital card to the analog card (T2T). This communication utilizes the eight trigger lines and employs a custom-written protocol to ensure an instruction communication rate of at least $\approx$5 MHz = 1/190 ns. $\mathbf{n}$ = photon counting registers, $\mathbf{t}_{\mathrm{d}}$ = detection times of the photons and the pulsed excitation, $\mathbf{r}_{\mathrm{s}}$ = scanning position.

the choice of localization estimators. Indeed, the tracking algorithm allows for independent update of the lateral $(x_{\mathrm{s}}, y_{\mathrm{s}})$ and axial (z_{s}) scanning positions. The current implementation relies only on the photon-counting registers $\mathbf{n}$ to check whether a new scanning position $\mathbf{r}_{\mathrm{s}}$ is required. Specifically, the photon countings are utilized to evaluate three main conditions: the presence of a particle inside the detection volume, the need for lateral re-centering, and the need for axial re-centering. In case it is needed, new hybrid modalities which consider the value of the fluorescence lifetime can easily be added.

The algorithm is structured into four main steps (Supplementary Fig. 11): collection, evaluation, decision and re-centering. Each step is executed at the cycle clock frequency of the logical unit $f_{\mathrm{c}} = 20$ MHz. After the initialization of the environment, in the "collection" step the algorithm reads the photon-counting registers $\mathbf{n}$ and updates the internal variables by integrating the number of photons detected since the

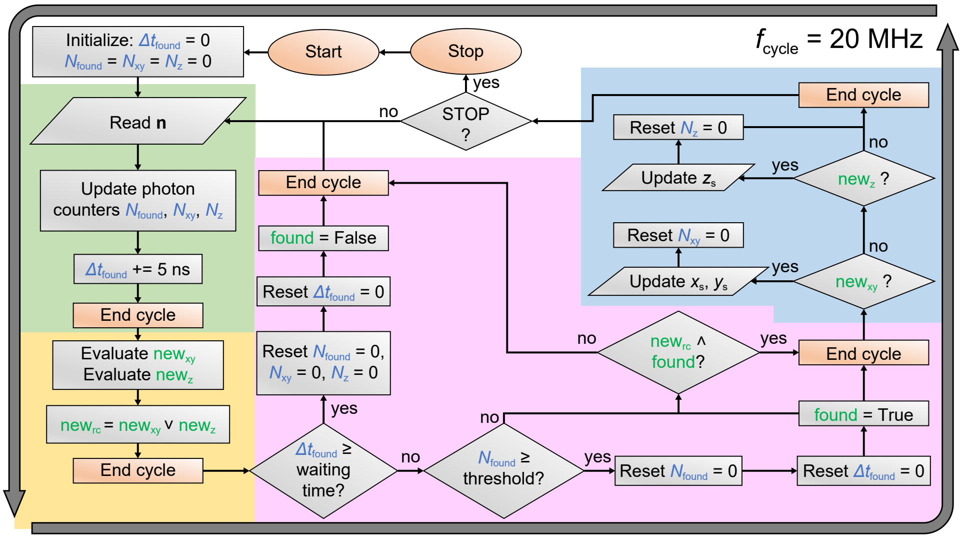

Supplementary Figure 11 Flowchart diagram of the tracking logical unit. The decision-making algorithm consists of four main steps, each represented by a different background color: green for "collection," yellow for "evaluation," pink for "decision," and blue for "re-centering." These steps are executed at a clock frequency of 20 MHz and are visually ended by an orange cycle breaking block. The "decision" step is the crucial junction in the algorithm, incorporating the necessary logical decisions to manage the real-time response of the system. It can lead to the subsequent "re-centering" step or loop back to the "collection" step. The algorithm utilizes both boolean variables (highlighted in green) and numeric variables (highlighted in blue). In the current implementation, the photon counting registers **n** are the only input.

last lateral (N_{xy}) and axial (N_{z}) re-centering events. In addition, it also keeps trace of the total amount of detected photons (N_{found}) and the elapsed time ($\Delta t_{\mathrm{found}}$) since the last determination of the presence of a particle.

In the subsequent "evaluation" step, the algorithm checks whether the user-defined conditions for the re-centering are satisfied, either in the lateral or axial direction. The user can choose between two alternative strategies: in the photon-counting mode, the evaluation is positive when the integrated registered photon-countings N_{xy} or N_{z} reach a specified threshold value, whereas in the fixed dwell time mode the triggering takes place at a predetermined rate, regardless of the number of photons.

When either the lateral or axial directions requires a re-centering, the scanning position does not immediately change. Instead, the system goes through the "decision" step, during which the update request is reviewed to ensure the photons are coming from a particle. To discriminate between a proper signal and the background, the system checks the values of N_{found} and $\Delta t_{\mathrm{found}}$ against a user-defined minimum signal photon flux. If the elapsed time exceeds the maximum waiting time without registering at least a minimum threshold amount of photons, then the data is labeled

as background and all the variables are reset. Consequently, the re-centering request is also discarded.

If the review is positive – the signal is considered valid – the process finally proceeds to the "re-centering" step, where a new scanning position is calculated.

Because of its dynamic real-time nature, the algorithm's execution time is not fixed and necessitates a minimum of 4 cycles, equivalent to 200 200 ns.

References

[1] Balzarotti, F. *et al.* Nanometer resolution imaging and tracking of fluorescent molecules with minimal photon fluxes. *Science* **355**, 606–612 (2017). URL https://www.science.org/doi/10.1126/science.aak9913. Publisher: American Association for the Advancement of Science.

[2] Masullo, L. A., Lopez, L. F. & Stefani, F. D. A common framework for single-molecule localization using sequential structured illumination. *Biophysical Reports* **2**, 100036 (2022). URL https://www.sciencedirect.com/science/article/pii/S2667074721000367.

[3] Sheppard, C. & Choudhury, A. Image Formation in the Scanning Microscope. *Optica Acta: International Journal of Optics* **24**, 1051–1073 (1977). URL https://doi.org/10.1080/713819421. Publisher: Taylor & Francis _eprint: https://doi.org/10.1080/713819421.

[4] Cox, I., Sheppard, C. & Wilson, T. Super-resolution by confocal fluorescent microscopy. *Optik - International Journal for Light and Electron Optics* **60**, 391–396 (1982).

[5] Rossi, M. & Kähler, C. J. Optimization of astigmatic particle tracking velocimeters. *Experiments in Fluids* **55**, 1809 (2014). URL https://doi.org/10.1007/s00348-014-1809-2.

[6] Volk, A. & Kähler, C. J. Density model for aqueous glycerol solutions. *Experiments in Fluids* **59**, 75 (2018). URL https://doi.org/10.1007/s00348-018-2527-y.

SUPPLEMENTARY INFORMATION FIGURES

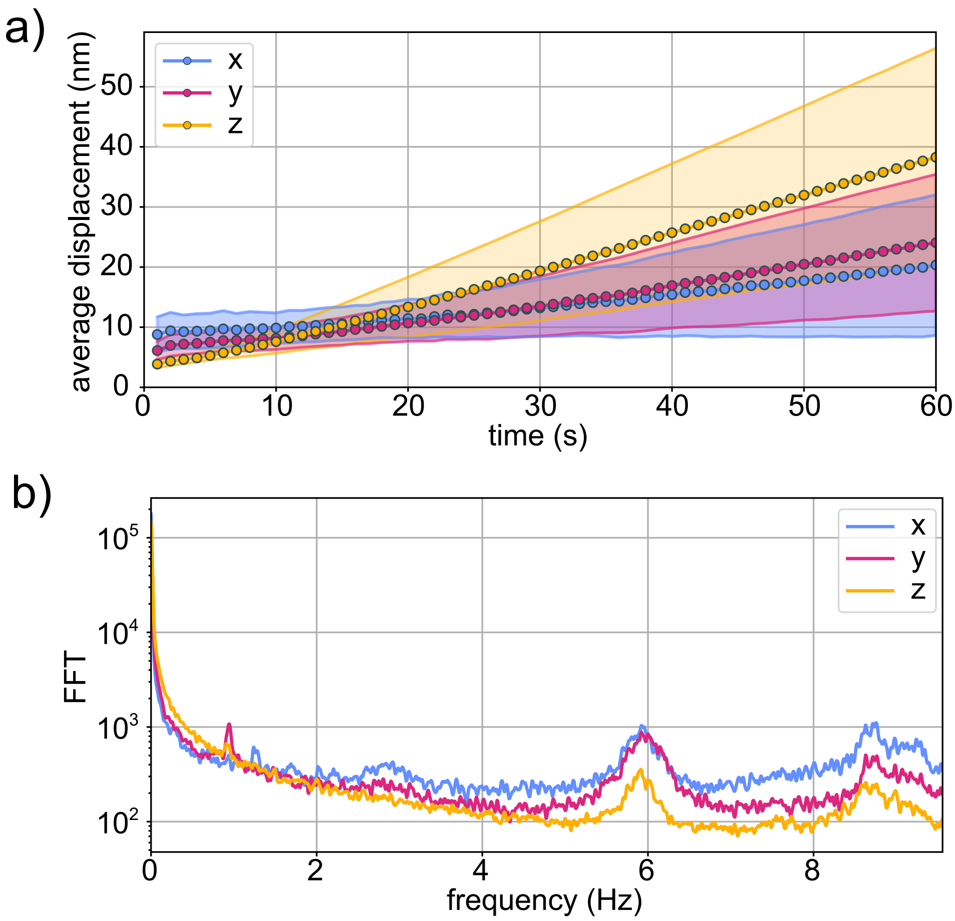

Supplementary Figure 12 Microscope stability during tracking. **a)** Average drift as a function of time. The curves are obtained by 5 independent experiments tracking 100 nm fluorescent beads ($\lambda_{\mathrm{exc}} = 488\,\mathrm{nm}$) in an immobile sample for more than 3 minutes. Each acquisition is performed with a fixed re-centering rate $\Delta t_{\mathrm{rc}}^{\mathrm{all}} = 50\,\mathrm{ms}$ at an average photon flux of $494 \pm 57\,\mathrm{kHz}$. The shaded area is the standard deviation. **b)** Average Fourier spectrum of the trajectories in **a**. The harmonic peaks of 6 Hz may be due to building oscillations not completely dumped by the optical table.

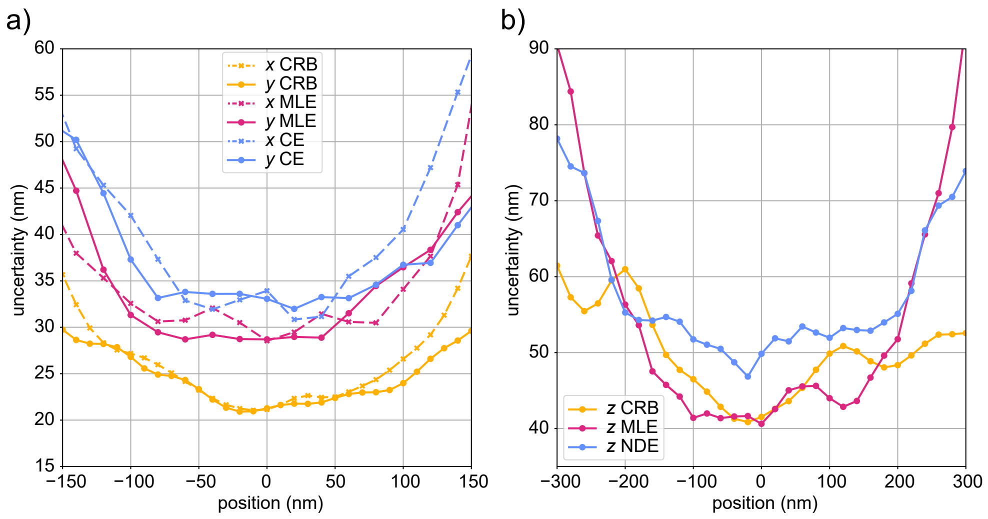

Supplementary Figure 13 Line profiles of the planar localization uncertainty maps. a) Line profiles of Fig. 2a-c along the x and y directions. **b)** Line profiles of Fig. 2d-f along the z direction.

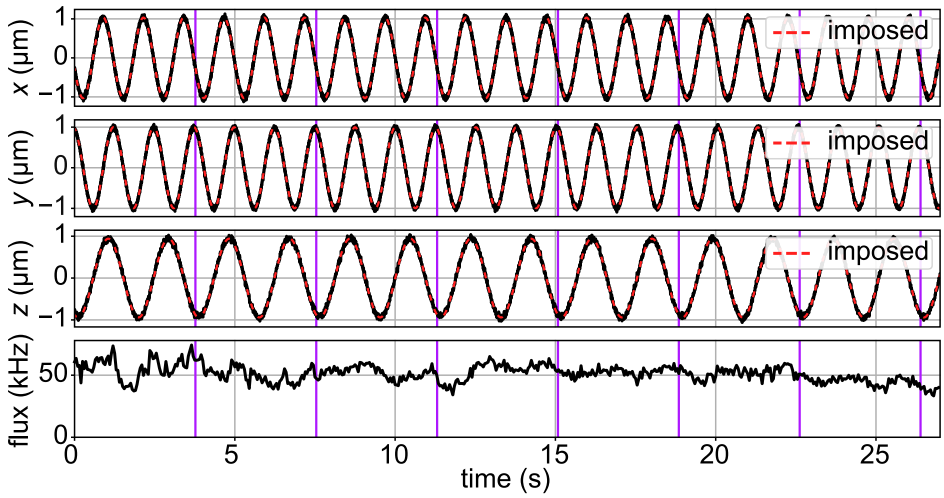

Supplementary Figure 14 Time evolution of the trajectory in Fig. 2g. The violet vertical lines mark the ending of each complete turn of the periodic pattern.

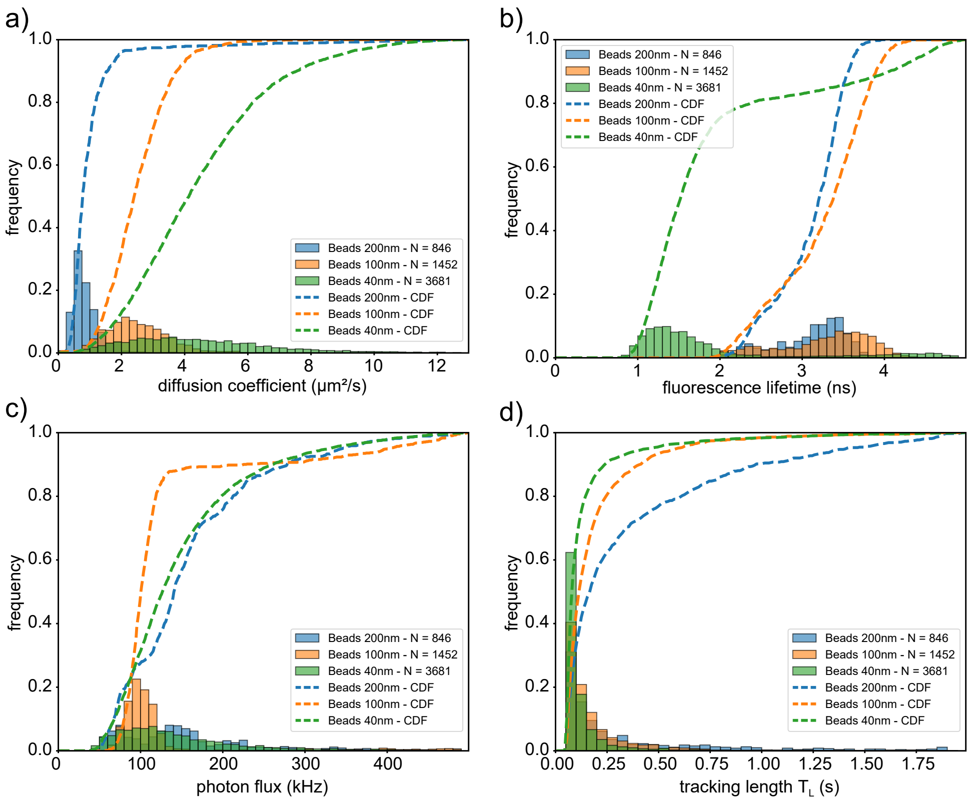

Supplementary Figure 15 Characterization of the 4D tracking dataset of freely diffusing fluorescent beads in water in Fig. 3a-d. a) Distributions of the measured diffusion coefficients. **b)** Distributions of the fluorescence lifetimes. **c)** Distributions of the photon flux. **d)** Distribution of the tracking time length. Each bead population is excited at $\lambda_{\mathrm{exc}} = 488\,\mathrm{nm}$. The legend reports the number of single trajectories acquired per bead size. For each single bead trajectory, the re-centering in the lateral and axial directions is performed at a fixed timing of $\Delta t_{\mathrm{rc}}^{\mathrm{lat}} = 1\,\mathrm{ms}$ and $\Delta t_{\mathrm{rc}}^{\mathrm{ax}} = 2\,\mathrm{ms}$ respectively.

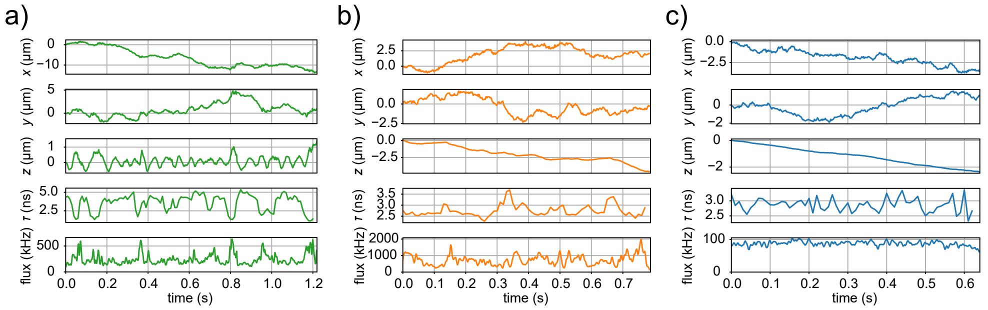

Supplementary Figure 16 Time evolution of the trajectories in Figs. 3c,d. a) 40 nm fluorescent bead. **b)** 100 nm fluorescent bead. **c)** 200 nm fluorescent bead.

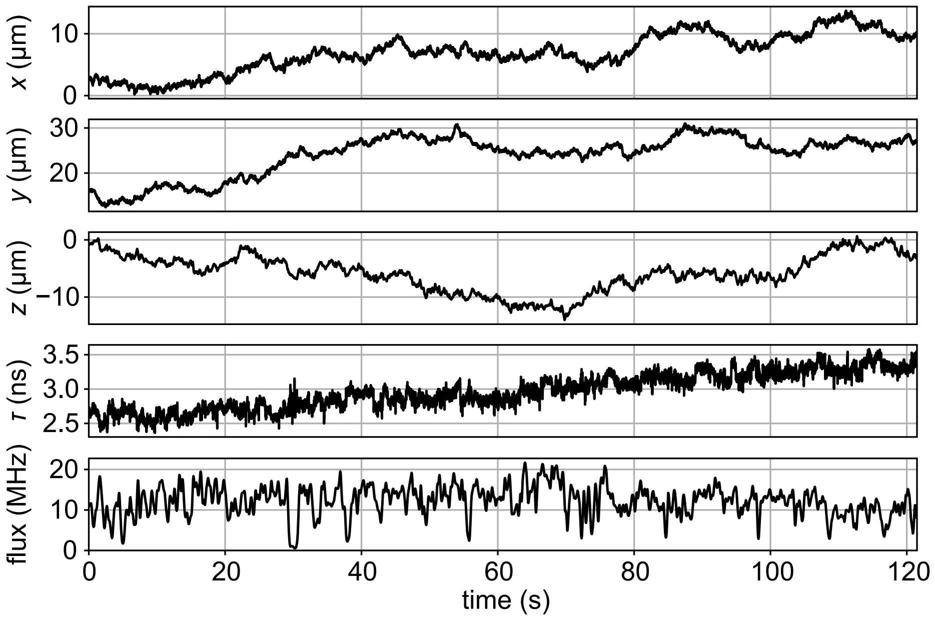

Supplementary Figure 17 Time evolution of the trajectory of Figs. 3e,f.

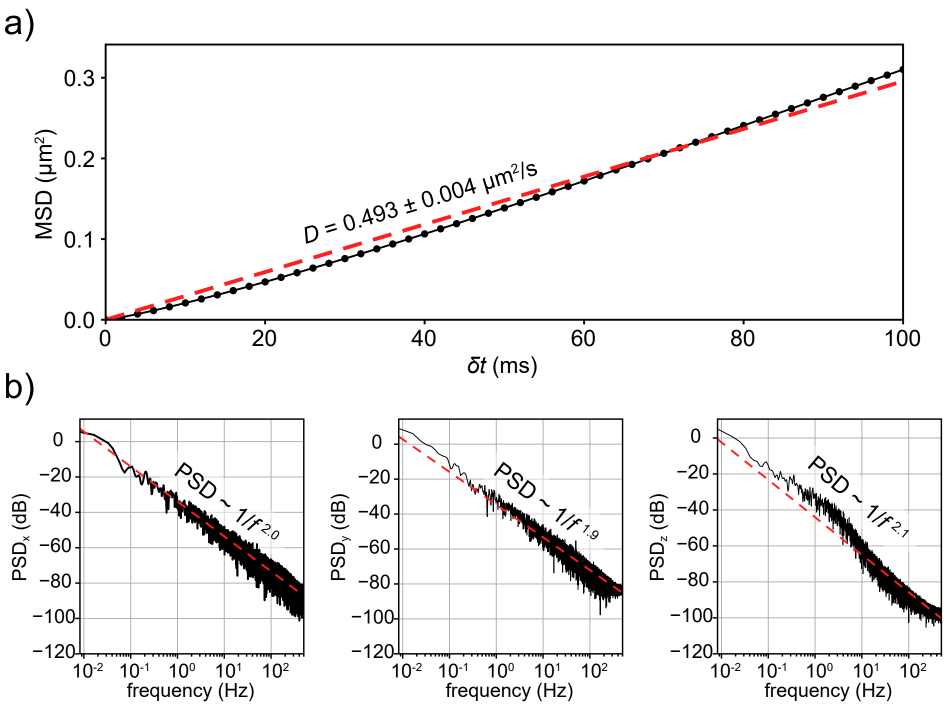

Supplementary Figure 18 Characterization of the motion regime of the trajectory in Figs. 3e,f. a) MSD of the trajectory with linear fitting indicating Brownian motion. **b)** Power spectral density of the trajectory along each spatial direction. The fitting reveals an exponential dependency over the frequency which is in good agreement with the one expected for pure Brownian motion ($\mathrm{PSD}(f) \sim 1/f^2$). The reference value for the dB scale is set to $1 \times 10^8\ \mathrm{nm}^2/\mathrm{Hz}$.

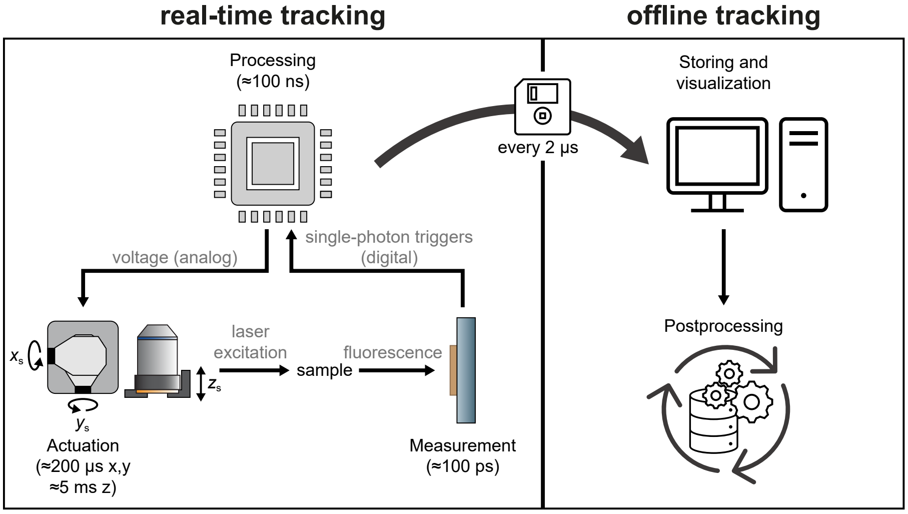

Supplementary Figure 19 Overview of the main stages of the tracking procedure. Our tracking approach features both a real-time feedback loop and an offline part. The feedback loop is composed of three cyclic steps: measurement, processing and actuation, with the latter currently representing the real-time speed bottleneck. The offline part relies on saved data and can be used to postprocess the trajectories to achieve higher temporal resolution ("rebinning") and lower spatial uncertainty ("refining").

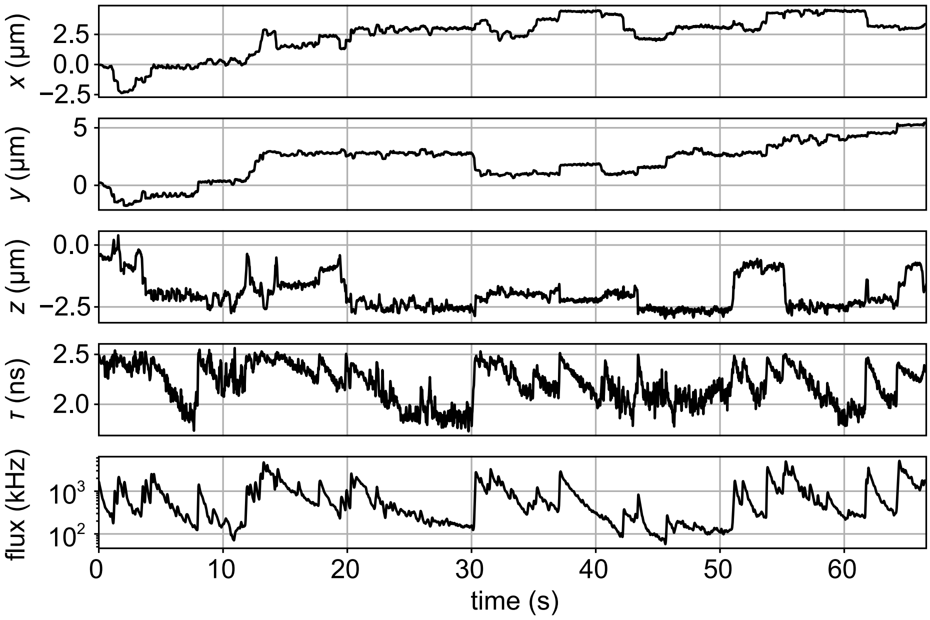

Supplementary Figure 20 Time evolution of the trajectory of Figs. 4a,b,d,e,f.

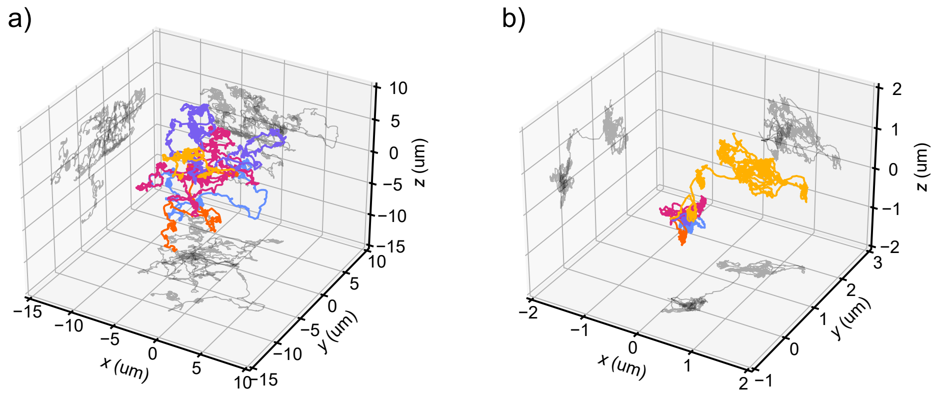

Supplementary Figure 21 3D trajectories of the lysosomes before and after the addition of nocodazole. **a)** 3D plot of 5 example trajectories of lysosomes moving inside living human SK-N-BE cells. **b)** 3D plot of 5 example trajectories of lysosomes moving in the same sample of **a** after the addition of nocodazole. All the measurements are performed with a fixed re-centering time $\Delta t_{\mathrm{rc}}^{\mathrm{all}} = 2.5\,\mathrm{ms}$.

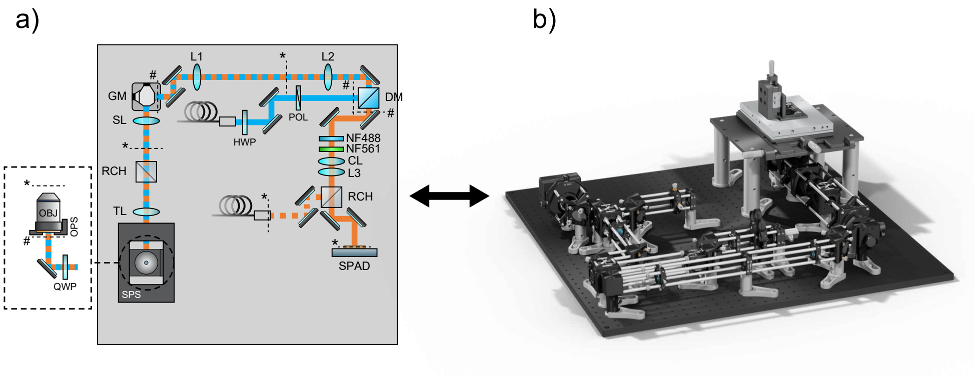

Supplementary Figure 22 Microscope setup. **a)** 2D sketch of the optical beam path. OBJ = objective, OPS = objective piezoelectric stage, SPS = sample piezoelectric stage, TL = tube lens, SL = scan lens, GM = galvanometric mirrors, DM = dichroic mirror, L1 = 200 mm, L2 = 150 mm, L3 = 300 mm, CL = cylindrical lens (f = 1000 mm), POL = polarizer, QWP = quarter-wave plate, HWP = half-wave plate, RCH = removable cube holder, NF488 = notch filter @ 488 nm, NF561 = notch filter @ 561 nm, * = imaging plane, # = phase plane. **b)** 3D render of the microscope.

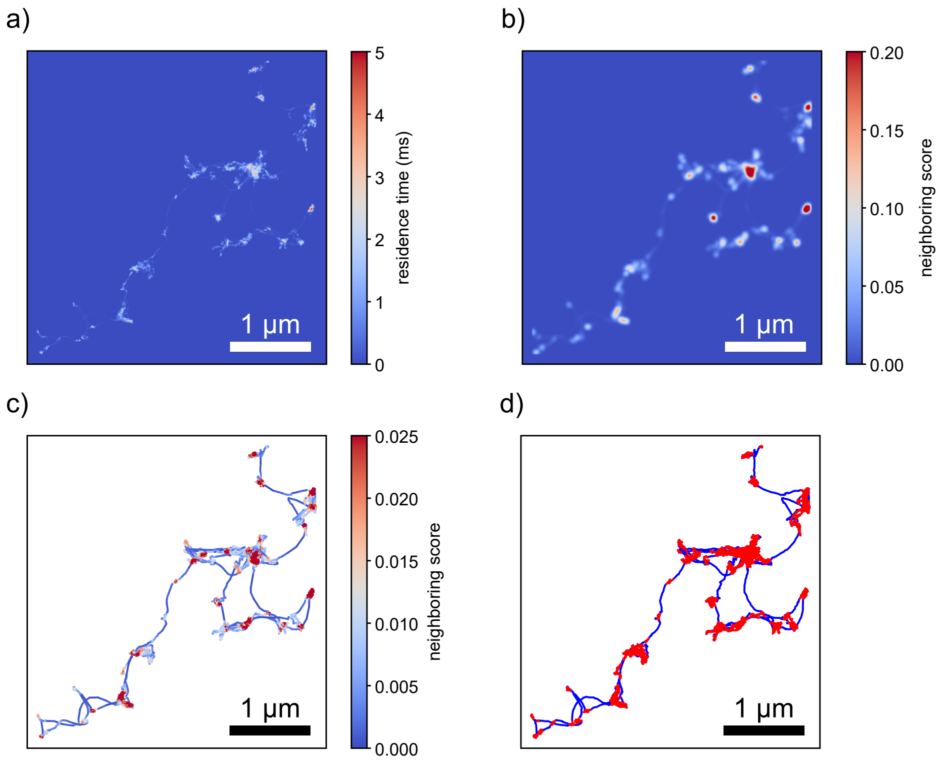

Supplementary Figure 23 Lysosome residence time and motion state segmentation. **a)** Maximum projection along the z axis of the voxel residence time histogram for the lysosome trajectory in Fig. 4. Voxel size is $5\,\text{nm}\times5\,\text{nm}\times20\,\text{nm}$. **b)** Maximum projection along the z axis of the neighboring score histogram. The 3D neighboring score is obtained by convolving the 3D histogram of the voxel residence time as in **a** with a Gaussian filter ($\sigma_x = \sigma_y = \sigma_z = 50\,\text{nm}$). **c)** 2D projection of the neighboring score of the lysosome trajectory. The graph is obtained by assigning at each trajectory point its corresponding neighboring score as calculated in **b**. **d)** 2D projection of the final segmented trajectory. The segmentation is obtained by thresholding the neighboring score in **c**: trajectory points with a score above 0.0125 are assigned to the "stop" class.

SUPPLEMENTARY INFORMATION TABLES

Diameter	λ_{exc}	Part number	Figure reference
20 nm	561 nm	Invitrogen™ FluoSpheres™ F8786	2, SF1
40 nm	561 nm	Invitrogen™ FluoSpheres™ F8793	2, SF3, SF6, SF8, SF13, SF14
20 nm	488 nm	Invitrogen™ FluoSpheres™ F8787	SF1
40 nm	488 nm	Invitrogen™ FluoSpheres™ F8795	3, SF15, SF16
100 nm	488 nm	Invitrogen™ FluoSpheres™ F8823	3, SF12, SF15, SF16
200 nm	488 nm	Invitrogen™ FluoSpheres™ F8811	3, SF15, SF16, SF17, SF18

Supplementary Table 1 List of all the fluorescent beads utilized in the various experiments.
