# Supplementary material for: 4D Single-particle tracking with asynchronous read-out single-photon avalanche diode array detector
Source: Nat Commun. 2024 Jul 23;15:6188. doi: 10.1038/s41467-024-50512-9 (PMC11266502; doi:10.1038/s41467-024-50512-9)
Supplement: Supplementary file 3 — Description of Additional Supplementary Files [file 41467_2024_50512_MOESM3_ESM.pdf]

Description of Additional Supplementary Files

Supplementary Movie 1: Video version of figure 4a

Supplementary Movie 2: Video version of figure 4f
