# Supplementary material for: 4D Single-particle tracking with asynchronous read-out single-photon avalanche diode array detector
Source: Nat Commun. 2024 Jul 23;15:6188. doi: 10.1038/s41467-024-50512-9 (PMC11266502; doi:10.1038/s41467-024-50512-9)
Supplement: Supplementary file 6 — Reporting Summary [file 41467_2024_50512_MOESM6_ESM.pdf]

nature portfolio

Corresponding author(s): Giuseppe Vicidomini
giuseppe.vicidomini@iit.it

Last updated by author(s): Jun 21, 2024

Reporting Summary

Nature Portfolio wishes to improve the reproducibility of the work that we publish. This form provides structure for consistency and transparency in reporting. For further information on Nature Portfolio policies, see our Editorial Policies and the Editorial Policy Checklist.

Statistics

For all statistical analyses, confirm that the following items are present in the figure legend, table legend, main text, or Methods section.

n/a	Confirmed	
☐	☒	The exact sample size (*n*) for each experimental group/condition, given as a discrete number and unit of measurement
☐	☒	A statement on whether measurements were taken from distinct samples or whether the same sample was measured repeatedly
☒	☐	The statistical test(s) used AND whether they are one- or two-sided<br>*Only common tests should be described solely by name; describe more complex techniques in the Methods section.*
☒	☐	A description of all covariates tested
☐	☒	A description of any assumptions or corrections, such as tests of normality and adjustment for multiple comparisons
☐	☒	A full description of the statistical parameters including central tendency (e.g. means) or other basic estimates (e.g. regression coefficient) AND variation (e.g. standard deviation) or associated estimates of uncertainty (e.g. confidence intervals)
☒	☐	For null hypothesis testing, the test statistic (e.g. *F*, *t*, *r*) with confidence intervals, effect sizes, degrees of freedom and *P* value noted<br>*Give P values as exact values whenever suitable.*
☒	☐	For Bayesian analysis, information on the choice of priors and Markov chain Monte Carlo settings
☒	☐	For hierarchical and complex designs, identification of the appropriate level for tests and full reporting of outcomes
☒	☐	Estimates of effect sizes (e.g. Cohen's *d*, Pearson's *r*), indicating how they were calculated

Our web collection on statistics for biologists contains articles on many of the points above.

Software and code

Policy information about availability of computer code

Data collection	Labview 2019
Data analysis	Python 3.7

For manuscripts utilizing custom algorithms or software that are central to the research but not yet described in published literature, software must be made available to editors and reviewers. We strongly encourage code deposition in a community repository (e.g. GitHub). See the Nature Portfolio guidelines for submitting code & software for further information.

Data

Policy information about availability of data

All manuscripts must include a data availability statement. This statement should provide the following information, where applicable:
- Accession codes, unique identifiers, or web links for publicly available datasets
- A description of any restrictions on data availability
- For clinical datasets or third party data, please ensure that the statement adheres to our policy

As keen proponents of open science and open data, the experimental data generated and analysed in this study are deposited in a publicly available Zenodo database.

Human research participants

Policy information about studies involving human research participants and Sex and Gender in Research.

Reporting on sex and gender	N/A
Population characteristics	N/A
Recruitment	N/A
Ethics oversight	N/A

Note that full information on the approval of the study protocol must also be provided in the manuscript.

Field-specific reporting

Please select the one below that is the best fit for your research. If you are not sure, read the appropriate sections before making your selection.

☒ Life sciences ☐ Behavioural & social sciences ☐ Ecological, evolutionary & environmental sciences

For a reference copy of the document with all sections, see nature.com/documents/nr-reporting-summary-flat.pdf

Life sciences study design

All studies must disclose on these points even when the disclosure is negative.

Sample size	In the tracking experiments that resulted in a single trajectory, we defined the sample size based on the number of localization points. For each specific particle of interest, we optimized the trajectory length by empirically adjusting the intensity flux to minimize photo-damage.<br>In the case of multiple trajectories experiments, such as the diffusion of beads of different size in water or in different water-glycerol solutions, we ensured a sufficiently high number of single particles (i.e., single trajectories) were acquired for each experiment to minimize statistical fluctuations when averaging the results. The specific sample sizes used are detailed in the manuscript.
Data exclusions	To filter out the false positives trajectory due to the system following a pattern on the detector caused by noise and not due to a single particle, we considered valid trajectories the ones which provided continuous localizations for at least 0.5 seconds. To minimize the impact of artifacts on the analysis of the fluorescent beads' diffusion in water-glycerol solutions, we applied a weighting procedure to the MSD curves of single particles. Each MSD curve was weighted based on its probability of belonging to a Gaussian distribution. The distribution parameters (mean, and standard deviation) were inferred from the overall unweighted population.<br>Other experiments didn't involve data exclusion.
Replication	Regarding the measurement of uncertainty maps, the experiment was replicated multiple times using different beads, and each replication consistently yielded the same result. The same applies to the assessment of the tracking uncertainties, when a bead was moved along an imposed path.<br>To validate the correlation between the motion state of the lysosome and the fluorescence lifetime of the GFP marker, 15 other independent trajectories were analyzed for consistency, as reported in the supplementary material.<br>In the case of other experiments, replication is not a significant concern.
Randomization	Randomization is not relevant for all the methods in the paper as they do not rely on the statistical variation of the samples. Generally, we ensured that the same particle was not measured twice, and during lysosome live cell experiments, we randomly changed the target cell before each acquisition.
Blinding	Blinding was ensured by analyzing all acquired data in a uniform manner. Any manual adjustments made during the analysis were consistently applied to all datasets.

Reporting for specific materials, systems and methods

We require information from authors about some types of materials, experimental systems and methods used in many studies. Here, indicate whether each material, system or method listed is relevant to your study. If you are not sure if a list item applies to your research, read the appropriate section before selecting a response.

Materials & experimental systems

n/a	Involved in the study
☒	☐ Antibodies
☐	☒ Eukaryotic cell lines
☒	☐ Palaeontology and archaeology
☒	☐ Animals and other organisms
☒	☐ Clinical data
☒	☐ Dual use research of concern

Methods

n/a	Involved in the study
☒	☐ ChIP-seq
☒	☐ Flow cytometry
☒	☐ MRI-based neuroimaging

Eukaryotic cell lines

Policy information about cell lines and Sex and Gender in Research

Cell line source(s)	SK-N-BE(2) are commercial cell line.
Authentication	SK-N-BE(2) cells were not authenticated.
Mycoplasma contamination	SK-N-BE(2) cells were routinely tested negative for mycoplasma contamination by PCR assay and via DAPI nuclear staining.
Commonly misidentified lines (See ICLAC register)	SK-N-BE(2) is not included in ICLAC register as commonly misidentified line.
